# Renal Lipid Alterations From Diabetes to Early‐Stage Diabetic Kidney Disease and Mitophagy: Focus on Cardiolipin

**DOI:** 10.1111/jcmm.70419

**Published:** 2025-02-12

**Authors:** Zhijie Li, Hongmiao Wang, Nan Liu, Xiayuchen Lan, Ailing Xie, Ge Yuan, Bowen Li, Jiaxin Geng, Xiaodan Liu

**Affiliations:** ^1^ Department of Nephrology The First Hospital of China Medical University Shenyang Liaoning China; ^2^ LipidALL Technologies Company Limited Changzhou Jiangsu China

**Keywords:** cardiolipin, cardiolipin synthase, diabetic kidney disease, free fatty acid, lipidomics, mitophagy, proximal tubule, sphingolipid, Szeto‐Schiller 31

## Abstract

Lipotoxicity plays a crucial role in the progression of diabetic kidney disease (DKD), yet the dynamic changes in renal lipid composition from diabetes to early‐stage DKD remain unclear. Free fatty acids, lactosylceramides and cardiolipin (CL) were identified as the most significantly altered lipids by quantitatively comparing targeted lipids in the renal cortex of the classic spontaneous diabetic *db/db* mice using high‐coverage targeted lipidomics. Further investigation into the causes and effects of decreased CL, which is a unique mitochondrial phospholipid, was conducted in mitochondria‐rich renal proximal tubular cells by using western blotting, real‐time PCR, immunohistochemistry and transmission electron microscopy. Reduced expression of cardiolipin synthase, a key enzyme in the CL synthesis pathway, and inhibition of CL‐related mitophagy were confirmed under high glucose conditions. In addition, the protective effect of CL‐targeted Szeto‐Schiller 31 in preserving mitophagy was demonstrated in both in vivo and in vitro studies. These findings provide new insights into the pathogenesis of early‐stage DKD from a lipid perspective and offer a theoretical basis for discovering new treatments.

**Abbreviations**
CDS: cytidine diphosphate diacylglycerol synthase
Cer: ceramides
CL: cardiolipin
CLS: cardiolipin synthase
DAG: diacylglycerols
DKD: diabetic kidney disease
FFA: free fatty acids
GalCer: galactosylceramides
GluCer: glucosylceramides
GM3: monosialodihexosyl ganglioside
HG: high glucose
HK‐2 cell: human renal proximal tubular epithelial cell
LacCer: lactosylceramides
MPC‐5 cell: podocyte Clone‐5 cell
NG: normal glucose
PGS: phosphatidylglycerol synthase
SS‐31: Szeto‐Schiller 31
TAG: triacylglycerols
TAZ: tafazzin
TC: cholesterol
TG: triglyceride
UACR: urine albumin‐to‐creatinine ratio

## 1 Introduction

Diabetic kidney disease (DKD) is a major cause of end‐stage renal disease, imposing substantial medical costs on society and significant burdens on families. Currently, beyond strict blood glucose control, the use of renin‐angiotensin‐system inhibitors and sodium‐glucose transporter 2 inhibitors, there are no effective treatments to delay the progression of DKD. Thus, elucidating the early pathogenesis of DKD and seeking new effective treatments remain urgent issues [1, 2, 3].

Emerging evidence supported that the proximal tubule may play a role as an initiator or contributor in the early pathogenesis of DKD. The proximal tubule is responsible for reabsorbing most of what is filtered, and, therefore, the proximal tubule has one of the highest mitochondrial contents in the body. Even in the early stage of diabetic kidney injury, mitochondrial fragmentation has been observed in proximal renal tubular epithelial cells. Mitophagy is a highly conserved process that selectively removes damaged or unnecessary mitochondria via the autophagic machinery, and its damage may play a fundamental role in the early pathogenesis of DKD [4, 5, 6, 7].

Lipotoxicity is a significant pathological and physiological factor in DKD. Hyperglycaemia‐induced abnormal lipid deposition and composition in renal intrinsic cells initiate various pathogenic mechanisms [8, 9, 10]. Lipids, such as cardiolipin (CL), have been identified to relocate the mitochondrial outer membrane under mitochondrial stress and interact with light chain 3 (LC3) directly to facilitate the recruitment of autophagosomes to initiate mitophagy [11]. However, the role and mechanism of lipid‐based mitophagy in renal damage in diabetes remain largely understudied.

Fully understanding the early changes in renal lipid composition is crucial for comprehending the mechanism of occurrence of DKD. In this study, lipid alterations from diabetes to early‐stage DKD were observed for the first time by quantitatively comparing the targeted lipids in the renal cortex of the classic spontaneous diabetic nephropathy animal model. Free fatty acids (FFA), lactosylceramides (LacCer) and CL were identified as the most significantly altered lipids. Focused on CL which is a unique mitochondrial phospholipid, further investigation into the reasons for the decrease in CL revealed a reduced expression of cardiolipin synthase (CLS), a key enzyme in the CL synthesis pathway, under high glucose (HG) conditions, and this was linked to damage to CL‐related mitophagy in proximal tubular cells. Additionally, the protective effect of mitochondrial CL‐targeted Szeto‐Schiller 31 (SS‐31) in preserving mitophagy was observed. These findings provide new insights into the early pathogenesis of DKD from a lipid perspective and offer a theoretical basis for discovering new treatments.

## 2 Materials and Methods

### 2.1 Animal Studies

All animal experiments were approved by the Institutional Animal Care and Use Committee of China Medical University. BKS.Cg‐^lepr^db/^lepr^db mice and BKS.Cg‐^lepr^db/+ mice, each group has 20 mice, were purchased from the Institute of Model Animals of Nanjing University and housed in a temperature‐controlled and humidity‐controlled specific‐pathogen‐free (SPF) barrier system in the Laboratory Animal Centre of China Medical University, with a 12‐h light/12‐h dark cycle. From the 12th week, the *db/db* mice were administered Elamipretide (Szeto‐Schiller‐31, SS‐31, Topscience, Shanghai, China) at a concentration of 3 mg/kg/day by intragastric administration for 8 weeks. The mice were allowed to eat and drink freely. They were fasted for 2 h prior to euthanasia. Urine, plasma, and kidney cortex samples were collected, snap‐frozen and stored at −80°C or preserved in paraformaldehyde, at 8, 12 and 20 weeks of age, respectively.

Quantification of albuminuria was performed by determining the urine albumin‐to‐creatinine ratio (UACR). Urinary albumin and creatinine levels were assessed using an ELISA assay and a commercial assay kit, respectively, both provided by Jiancheng Biotech Co. Ltd. (Nanjing, China). Serum triglycerides (TG) and total cholesterol (TC) were measured in accordance with the manufacturer's instructions. Prior to tissue collection, the kidneys were perfused to eliminate potential blood interference. Sections of 3 μm thickness from paraformaldehyde‐fixed, paraffin‐embedded kidney slices were stained using Periodic Acid‐Schiff's (PAS) reagent.

### 2.2 Lipid Extraction and Lipidomics Analysis

Lipids were extracted from approximately 30 mg of frozen tissue using a modified version of the Bligh and Dyer method, as previously described [12]. Lipidomic analyses were conducted using an ExionLC‐AD coupled with a Sciex QTRAP 6500 PLUS at LipidALL Technologies Company Limited [13, 14, 15]. A detailed description is provided in the Supporting Information.

### 2.3 Cell Culture and Treatment

A detailed description of cell culture, as outlined in the team's previous work, is provided in the Supporting Information [16]. Human renal proximal tubular epithelial cells (HK‐2 cells) and the mouse podocyte Clone‐5 cells (MPC‐5 cells) were divided into the following groups: normal glucose group (NG, 5.6 mmol/L glucose), high glucose group (HG, 30 mmol/L glucose) and SS‐31 groups treated with SS‐31 at a concentration of 100 nM for 48 h in 5.6 mmol/L glucose (NG‐SS‐31) and 30 mmol/L glucose (HG‐SS‐31), respectively.

### 2.4 Cell Transfection

To overexpress CLS, a CLS overexpression pcDNA3.1(+) plasmid was constructed using the mouse CLS protein sequence obtained from a previous study. CLS overexpression was verified by western blotting following transfection, and the overexpression plasmid was used in subsequent experiments. Various CLS knockdown siRNAs were transfected into recipient cells using Lipo8000 Transfection Reagent (Beyotime, Shanghai, China) according to the manufacturer's instructions. Western blotting and RT‐qPCR were employed to identify the siRNA with the highest knockdown efficiency for use in subsequent studies.

### 2.5 Western Blotting

A detailed description of Western blotting, as outlined in the team's previous work, is provided in the Supporting Information [16]. The primary antibodies used included anti‐LC3 Rabbit Antibody (1:1000, Abmart, Shanghai, China), anti‐P62 Rabbit Antibody (1:1000, Affinity Biosciences, USA), anti‐CLS Rabbit Antibody (1:1000, Affinity Biosciences, USA), anti‐phosphatidylglycerol synthase (PGS) Rabbit Antibody (1:500, Abcam, Cambridge, UK), anti‐cytidine diphosphate diacylglycerol synthase (CDS) Rabbit Antibody (1:1000, Abcam, Cambridge, UK) and anti‐tafazzin (TAZ) Rabbit Antibody (1:1000, Abcam, Cambridge, UK). HRP‐labelled Goat Anti‐Rabbit IgG (1:30,000, Earthox, San Francisco, USA) and Horseradish enzyme‐labelled Goat Anti‐Mouse IgG (1:30,000, Earthox, San Francisco, USA) were used as secondary antibodies. Specific bands were detected using an enhanced chemiluminescent substrate (Beyotime, Shanghai, China). β‐Actin Mouse Monoclonal Antibody (1:1000, Abmart, Shanghai, China) or GAPDH Mouse Monoclonal Antibody (1:1000, Abmart, Shanghai, China) was used as the internal control. Relative band intensity was measured using ImageJ software.

### 2.6 Real‐Time PCR

Total RNA of CLS, CDS, PGS and TAZ was extracted using TRIzol reagent (Invitrogen, Carlsbad, CA, USA). The concentration of the collected RNA was measured by spectrophotometry. cDNA was then synthesised using a reverse transcription kit (TaKaRa, Japan). mRNA expression levels were determined using a QuantStudio 1 Real‐Time PCR Instrument (Thermo, Massachusetts, USA). GAPDH was used as a control for normalisation.

### 2.7 Isolation of HK‐2 Cells Mitochondria

Mitochondria were isolated by differential centrifugation as follows. When the number of HK‐2 cells reached (2–5) × 10^7^, the original medium was discarded, and the cells were washed with PBS and digested with trypsin. The cells were then collected by centrifugation at 200 *g* for 10 min. After re‐suspending the cells in PBS, they were centrifuged at 600 *g* at 4°C for 5 min. The supernatant was discarded, and mitochondrial separation solution (containing 1 mM PMSF) was added to the tube. The cells were treated in an ice bath for 15 min and homogenised 10–15 times until the trypan blue test, observed under a microscope, met the required standard. Differential centrifugation was performed first at 600 *g* at 4°C for 10 min, followed by centrifugation of the supernatant at 11,000 *g* at 4°C for 10 min. The mitochondrial pellet was collected from the bottom of the tube.

### 2.8 Measurement of Total Cardiolipin Concentration in Cells

The total CL concentration in HK‐2 cells and podocytes was measured using ELISA kits (Andygene, Beijing, China), following the manufacturer's instructions.

### 2.9 Transmission Electron Microscopy

The kidney tissues and centrifuged cell pellets were pre‐fixed with 2.5% glutaraldehyde overnight at 4°C and post‐fixed samples with 10 g/L osmium tetroxide at 4°C for 2 h. Samples were washed with distilled water three times for 10 min and dehyrated with 30%, 50%, 70%, 90% and 100% alcohol and 100% propylene oxide for 5 min each. Samples were infiltrated by 1/2 epoxy resin and pure epoxy resin for 1 h each and polymerised at 60°C for 24 h. Ultramicrotome (Leica UC7) was used to cut 70 nm ultrathin sections. Sections were stained with 30 g/L uranyl acetate for 20 min and 26 g/L lead citrate for 5 min. After drying the copper grid, transmission electron microscope (HITACHI, HT7700, Japan) was employed for the acquisition of images. The thickness of proximal tubular basement membrane was measured using ImageJ software.

### 2.10 Detection of the Mitochondrial Membrane Potential (MMP)

HK‐2 cells were seeded into 12‐well plates. After discarding the original medium, 50 μL of fresh medium was added, followed by 50 μL of JC‐1 staining working solution (JC‐1200X: ultrapure water: JC‐1 staining buffer 5× = 1:160:40). The plates were gently shaken to ensure adequate mixing and then incubated at 37°C for 20 min. The liquid was discarded, and the wells were washed twice with JC‐1 staining buffer (1×). Finally, 1 mL of fresh medium was added to each well, and fluorescence distribution was observed using an inverted fluorescence microscope.

### 2.11 Mitochondrial Adenosine Triphosphate (ATP) Assay

HK‐2 cells were cultured in six‐well plates. After discarding the original media and washing twice with PBS, all steps were performed on ice according to the kit instructions. Briefly, 200 μL of lysate was added to each well and incubated on ice. The supernatant was then collected by centrifugation using a low‐temperature centrifuge. Next, 100 μL of ATP detection working solution was added to the detection wells of a 96‐well plate with black side walls, and 20 μL of the sample was added to each well, except for the blank wells. The relative light unit (RLU) values were measured using a chemiluminescence method. A standard curve was created based on concentration and RLU values to calculate the ATP concentration of the samples. The protein concentration of the samples was determined using the BCA assay, and the ATP value per protein concentration was used as the standard for analysis and comparison.

### 2.12 Immunohistochemistry

Paraffin‐embedded kidney slices (7‐μm thick) were deparaffinised, rehydrated and subjected to antigen retrieval. Then, the slides were incubated with anti‐CLS Rabbit Antibody (1:100, Affinity Biosciences, USA) overnight at 4°C, and subsequently incubated with the secondary antibody (ZSGB‐BIO, Beijing, China). DAB stain was used, followed by haematoxylin counterstaining. Finally, a microscope (Nikon, Japan) was used for image capture. Each animal randomly selected four fields to measure using ImageJ software for quantitative analysis.

### 2.13 Statistical Analysis

Principal component analysis (PCA) was conducted on centred and scaled values using the R package ‘FactoMineR’ [17]. Orthogonal partial least squares discriminant analysis was performed using the R package ‘ropls’ [18]. Overfitting was assessed by pR^2^Y and pQ^2^ derived from permutation analysis (20 times) with seven‐fold cross‐validation. Hierarchical clustering with complete linkage was visualised using the R package ‘ComplexHeatmap’. Differential analysis involving more than two groups was conducted using parametric Welch's ANOVA test with a post hoc Games‐Howell test. Differential analysis between two groups was performed using a parametric t‐test. All data are presented as mean ± SEM, and *p* < 0.05 was considered statistically significant. Statistical analyses were carried out using R 4.3.3 and SPSS 26.0 software.

## 3 Results

### 3.1 Characteristics of Diabetic *db/db* Mice

**FIGURE 1:**
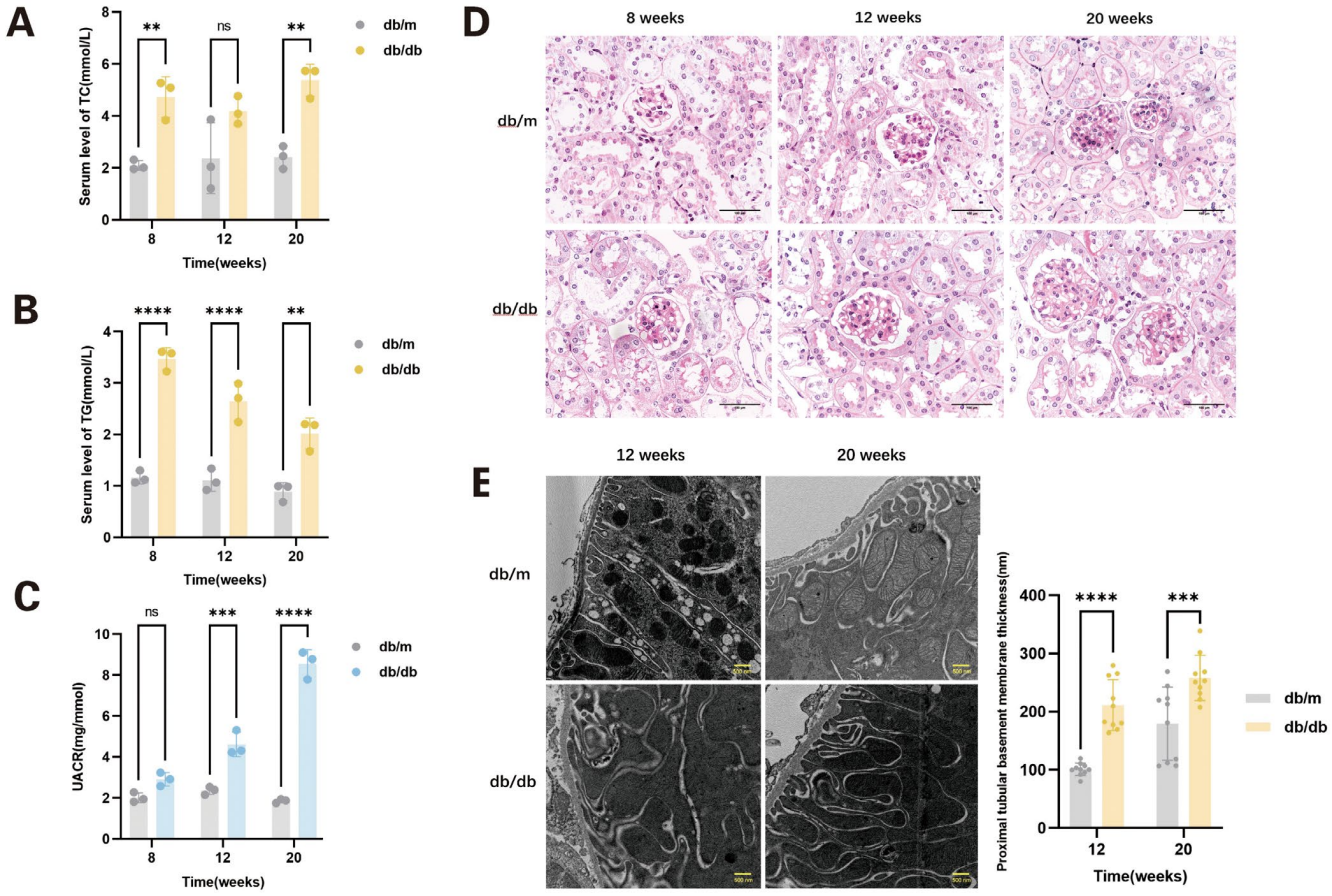
Characteristics of the diabetic *db/db* mice and wild‐type *db/m* mice at various ages. (A) Serum total cholesterol (TC); (B) serum triglyceride (TG); (C) urine albumin‐to‐creatinine ratio (UACR); (D) periodic acid‐Schiff's (PAS) staining (magnification: 400×). (E) Comparison of proximal tubular basement membrane thickness by transmission electron microscopy (magnification: 20,000×). Data are presented as means ± SEM, *n* = 3 per group; **p < 0.01, ***p < 0.001, ****p < 0.0001.

At 8 weeks of age, the glucose levels in *db/db* mice were 16.7 ± 4.6 mmol/L, indicating hyperglycaemia. In both the 8‐week and 20‐week *db/db* mice groups, serum TC levels were notably elevated. Additionally, at 8, 12 and 20 weeks of age, the serum TG levels in *db/db* mice were significantly higher compared to those in control *db/m* mice (Figure 1A,B). UACR began to increase in *db/db* mice starting at 12 weeks of age (Figure 1C). Compared to 8‐week *db/db* mice and 12‐week *db/m* mice, increased glomerular size and mild mesangial expansion, as observed through PAS staining, were noted in 12‐week *db/db* mice, with both conditions exacerbated in 20‐week *db/db* mice, along with mild interstitial oedema (Figure 1D). Starting from 12 weeks of age, the basement membrane of the proximal tubules in *db/db* mice was significantly thickened compared to the control group mice (Figure 1E), and the swelling cristae of mitochondria were observed in some proximal tubular cells by transmission electron microscope (Figure S1).

### 3.2 Differential Expression of Renal Cortex Lipids

To assess the lipid composition and differential expression profiles of the renal cortex, a high‐coverage and targeted lipidomic analysis was performed. In this study, reproducibility of the lipidomics run was confirmed by quality control (QC) samples (aliquots of pooled sample) injected between actual samples during the MS run. The four QC samples were clustered together at the centre of the PCA score plots, indicating stability and reproducibility of the MS analysis (Figure S2). In the renal cortex, 30 major lipid classes comprising 728 species were identified, with triacylglycerides (TAG) accounting for the highest number of species (119) (Figure S3).

**FIGURE 2:**
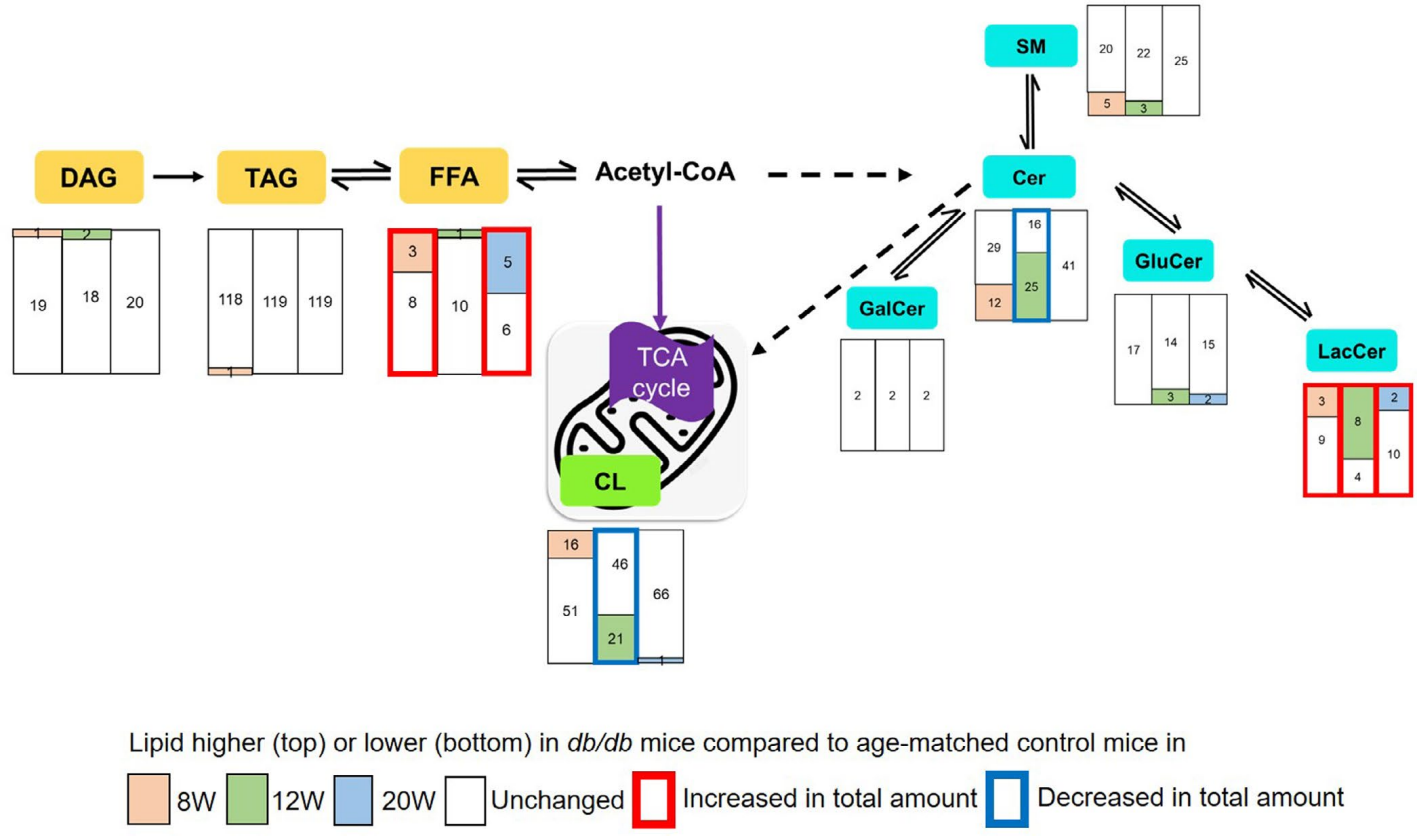
Differential expression of renal cortex lipids between age‐matched diabetic *db/db* mice and wild‐type *db/m* mice at 8, 12 and 20 weeks. The numbers in the box indicate the number of detected lipids. Cer, ceramides; CL, cardiolipin; DAG, diacylglycerols; FFA, free fatty acids; GalCer, galactosylceramides; GluCer, glucosylceramides; LacCer, lactosylceramides; SM, sphingomyelins; TAG, triacylglycerols; *n* = 3 per group.

The expression of lipids in the renal cortex was compared between age‐matched diabetic *db/db* mice and wild‐type *db/m* mice at 8, 12 and 20 weeks (Figure 2). The FFA, LacCer and CL were identified as the most significantly altered lipids. Total FFA were significantly increased in *db/db* mice at 8 and 20 weeks compared to age‐matched control mice. Among these, FFA 20:4, FFA 20:5, FFA 22:4, FFA 22:5 and FFA 22:6 exhibited the most significant changes (Figure S4). Among sphingolipids, total LacCer were significantly increased in *db/db* mice at 8, 12 and 20 weeks compared to age‐matched control mice, and LacCer d18:1/24:0, LacCer d18:0/16:0, LacCer d18:1/16:0 and LacCer d18:1/14:0 showed the most significant changes (Figure S5). Total ceramides (Cer) were significantly decreased in 12‐week *db/db* mice compared to 12‐week control mice (Figure S5). Glucosylceramides (GluCer) d18:1/21:0 and GluCer d18:1/18:0 were significantly decreased in 20‐week *db/db* mice compared to 20‐week control mice (Figure S5). There were no statistically significant differences in gangliosides between 20‐week *db/db* diabetic mice and *db/m* control mice (Figure S6).

Total CL was notably decreased in *db/db* mice at 12 weeks compared to age‐matched control mice, and the CL species showing the most significant changes included CL 78:11(18:2), CL 78:12(18:2), CL 78:13(18:2), CL 78:13(20:3), CL 78:13(20:4), CL 78:14(18:2), CL 76:10(20:4), CL 76:10(18:2), CL 76:11(18:2), CL 76:12(18:2), CL 74:7(18:2), CL 74:9(20:4), CL 74:9(18:2), CL 74:10(20:4), CL 74:10(18:2), CL 72:7(20:4), CL 72:7(18:2), CL 72:8(18:2), CL 70:4(18:2), CL 70:5(18:2), and CL 68:5(18:2). CL 78:13(20:3) was significantly increased in *db/db* mice at 8 weeks compared to age‐matched control mice but decreased in *db/db* mice at 12 weeks. CL 76:9(18:2) was decreased in both 12‐ and 20‐week *db/db* mice compared to corresponding age‐matched control mice (Figures S7 and S8).

### 3.3 High Glucose Reduces CL Expression by Decreasing CLS

**FIGURE 3:**
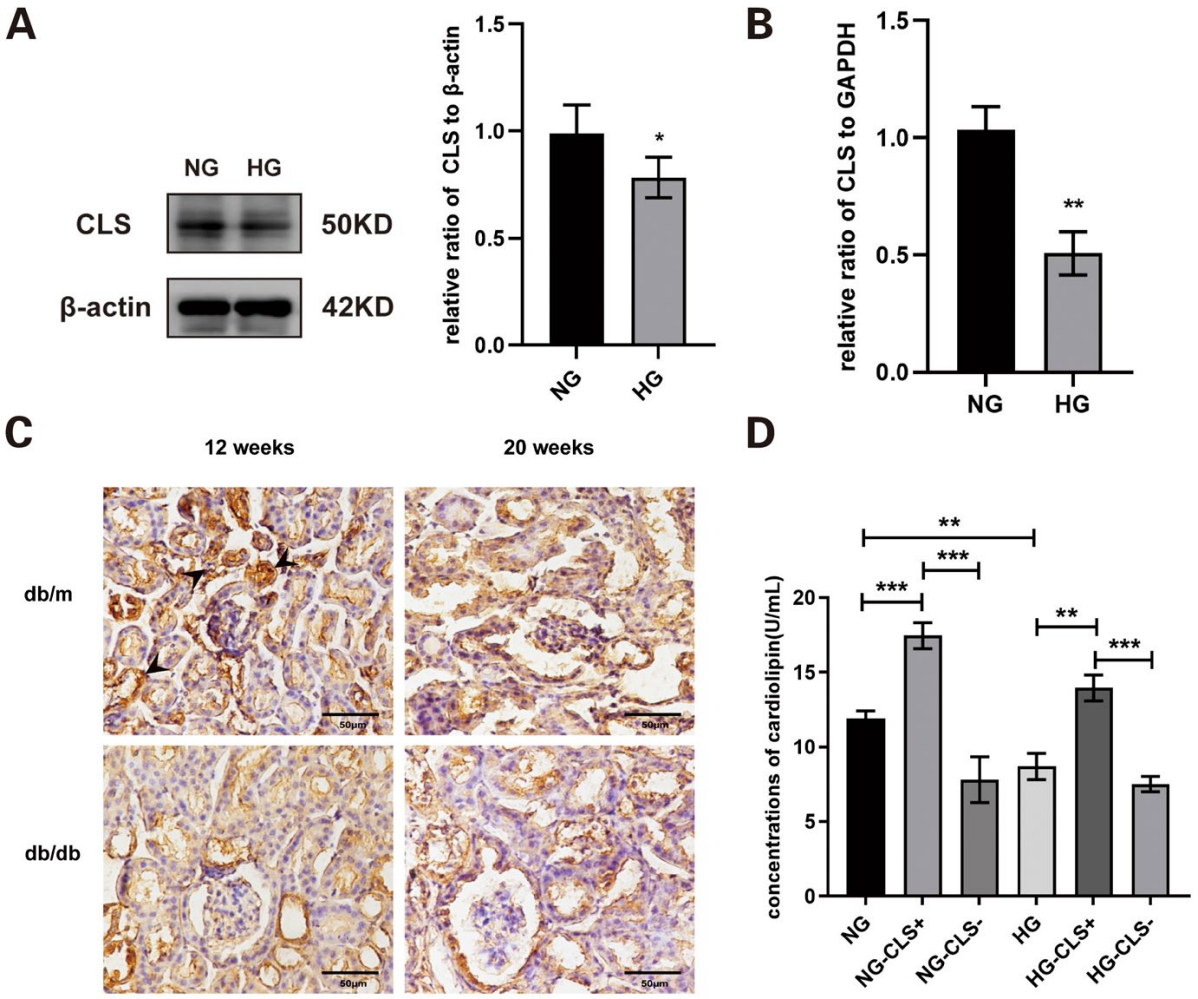
Effect of CLS on CL level in HK‐2 cells and the expression of CLS in renal cortex. (A) The protein level of CLS in HK‐2 cells was quantified using western blotting. (B) The mRNA expression of CLS in HK‐2 cells was assessed by RT‐PCR. (C) The expression of CLS was observed by immunohistochemistry (indicated by arrows). (D) The total level of CL in different groups was measured using ELISA. CL, cardiolipin; CLS, cardiolipin synthase; CLS−, CLS‐siRNA knockdown group; CLS+, CLS expression plasmid transfection group; HG, high glucose group; NG, normal glucose group; *n* = 3, *p < 0.05, **p < 0.01, ***p < 0.001.

To clarify the reason for the reduced expression of CL under high glucose stimulation, key enzymes involved in CL synthesis—CDS, PGS, CLS and TAZ—were assessed in HK‐2 cells. Both protein and mRNA expression levels of CLS were significantly reduced in the HG group (Figure 3A,B). No significant differences were observed in the protein expression levels of TAZ, PGS and CDS between the HG and NG groups. However, in the HG group, the mRNA expression of TAZ and PGS was notably decreased, while CDS mRNA expression showed no significant change (Figure S9). The immunohistochemical results showed that CLS was widely expressed in the renal cortex, especially in renal tubules, and the expression in *db/db* mice at 12 weeks of age showed a decreasing trend compared to the control group, but there was no statistical difference between the groups (Figure 3C).

To further explore how CLS affects CL expression in HK‐2 cells, CLS overexpression and knockdown treatments were performed, with efficacy validated by western blotting and RT‐PCR (Figure S10). Results indicated that CL levels in HK‐2 cells were decreased under high glucose conditions and with low CLS expression (Figure 3D).

### 3.4 Effect of CLS on Mitophagy, Mitochondrial Morphology and Function

**FIGURE 4:**
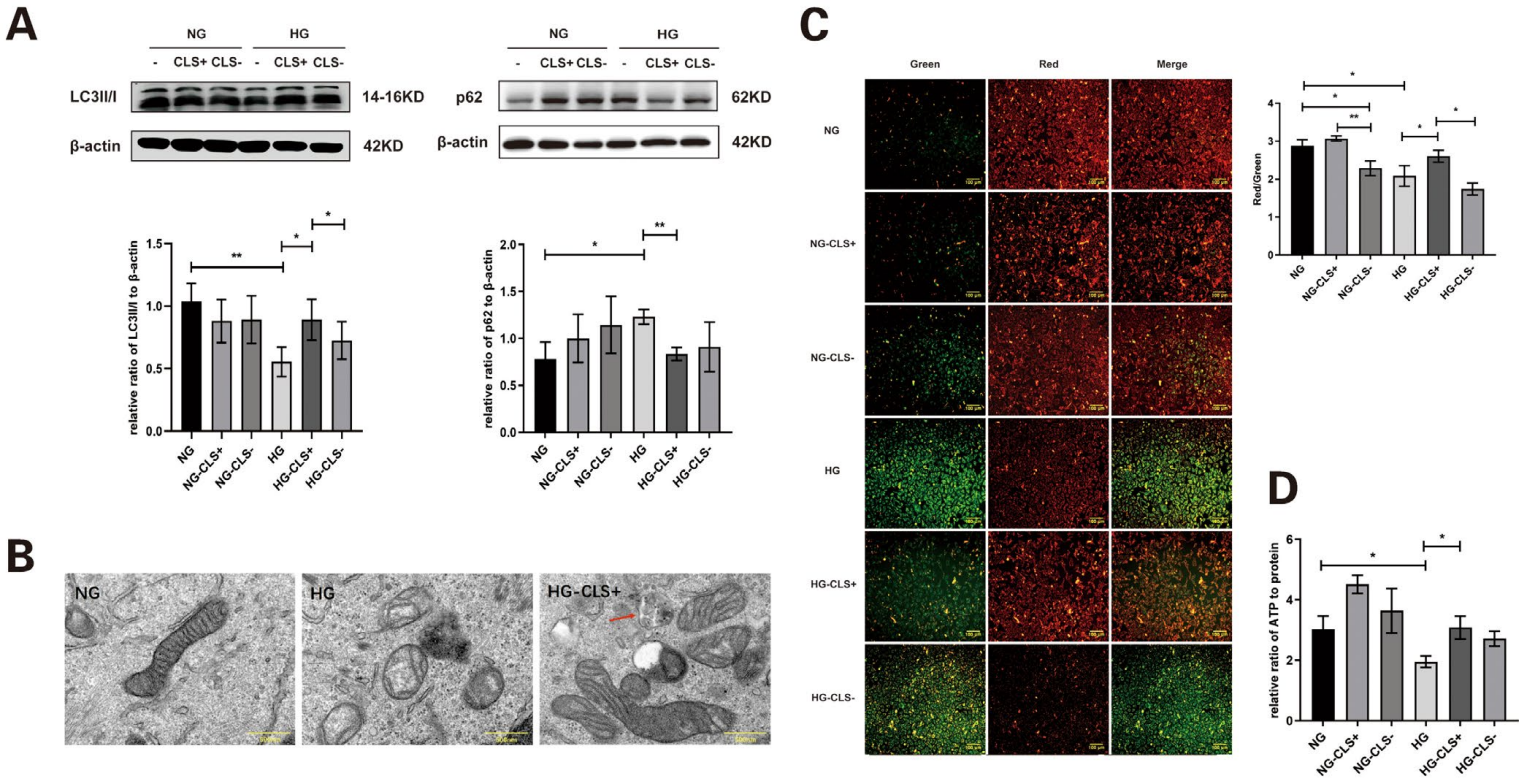
Effect of changes in CLS on mitophagy, membrane potential and ATP concentration of mitochondria in HK‐2 cells. (A) LC3II/I and p62 levels of mitochondrial protein extracted from HK‐2 cells were detected using western blotting. (B) Mitochondria and autophagosomes (indicated by red arrows) in HK‐2 cells were observed using transmission electron microscopy (magnification: 20,000×). (C) Mitochondrial membrane potential in HK‐2 cells was measured. (D) ATP concentration in HK‐2 cells was measured. CLS, cardiolipin synthase; CLS−, CLS‐siRNA knockdown group; CLS+, CLS expression plasmid transfection group; HG, high glucose group; NG, normal glucose group; *n* = 3, *p < 0.05, **p < 0.01.

Autophagy‐related mitochondrial proteins extracted from HK‐2 cells were assessed using western blotting. Compared to the NG group, the LC3II/I ratio was significantly lower in the HG group. However, LC3II/I notably increased in the HG group following CLS overexpression. Additionally, p62 levels were significantly elevated in the HG group but were substantially reduced with CLS overexpression in the HG group (Figure 4A).

In HK‐2 cells, transmission electron microscopy revealed swelling, fragmentation of mitochondria and disappearance of cristae in the HG group. In contrast, cells with CLS overexpression displayed largely intact mitochondrial morphology, with visible mitochondrial autophagosomes (Figure 4B).

The MMP was notably decreased in the HG and CLS knockout groups but was significantly increased in the CLS overexpression group (Figure 4C). Additionally, ATP concentration in HK‐2 cells decreased in the HG group, while it increased in the HG group with CLS overexpression (Figure 4D).

### 3.5 Protective Effect of SS‐31 on Mitochondria Through CL

**FIGURE 5:**
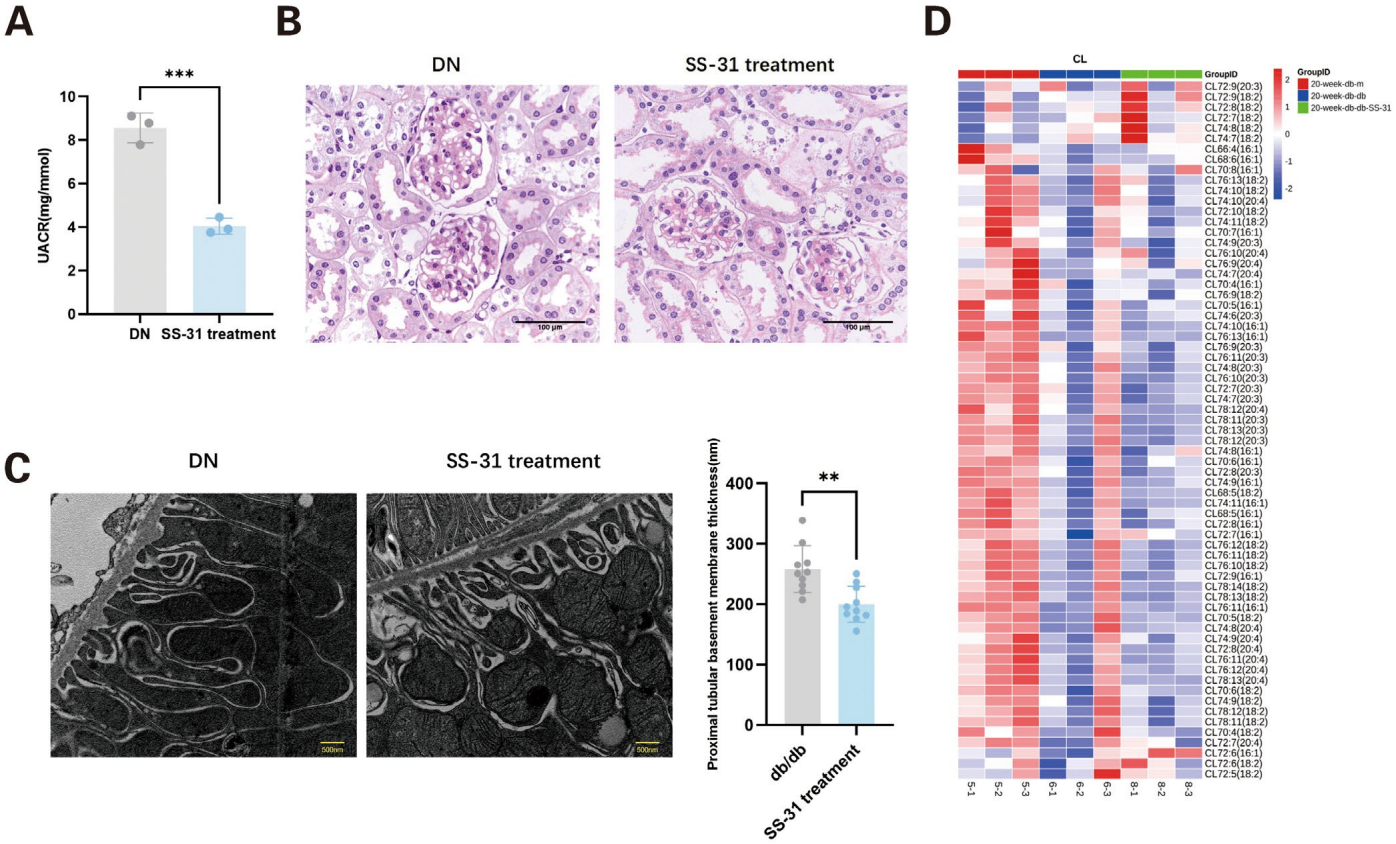
Effect of SS‐31 on DN mice. (A) Urine albumin‐to‐creatinine ratio (UACR). (B) Periodic acid‐Schiff's (PAS) staining (magnification: 400×). (C) Comparison of renal tubular basement membrane thickness by transmission electron microscopy (magnification: 20,000×). (D) Heatmap of cardiolipin (CL) in renal cortex. DN, diabetic nephropathy group (20‐week *db/db* mice); SS‐31 treatment, 20‐week *db/db* mice treated with SS‐31; SS‐31, Szeto‐Schiller 31; data are presented as means ± SEM, *n* = 3; **p < 0.01, ***p < 0.001.

In in vivo studies, compared to diabetic nephropathy (DN) mice, the UACR was significantly reduced in SS‐31‐treated mice (Figure 5A). Additionally, SS‐31 treatment resulted in decreased glomerular volume and reduced matrix deposition (Figure 5B). The thickness of basement membrane of proximal tubules in the SS‐31 treatment mice was significantly reduced compared to the DN mice, and the cristae and outer membrane of mitochondria were also observed to be clearer in SS‐31 group (Figure 5C). The total expression level of CL in renal cortex was not increased following SS‐31 treatment (Figure 5D).

**FIGURE 6:**
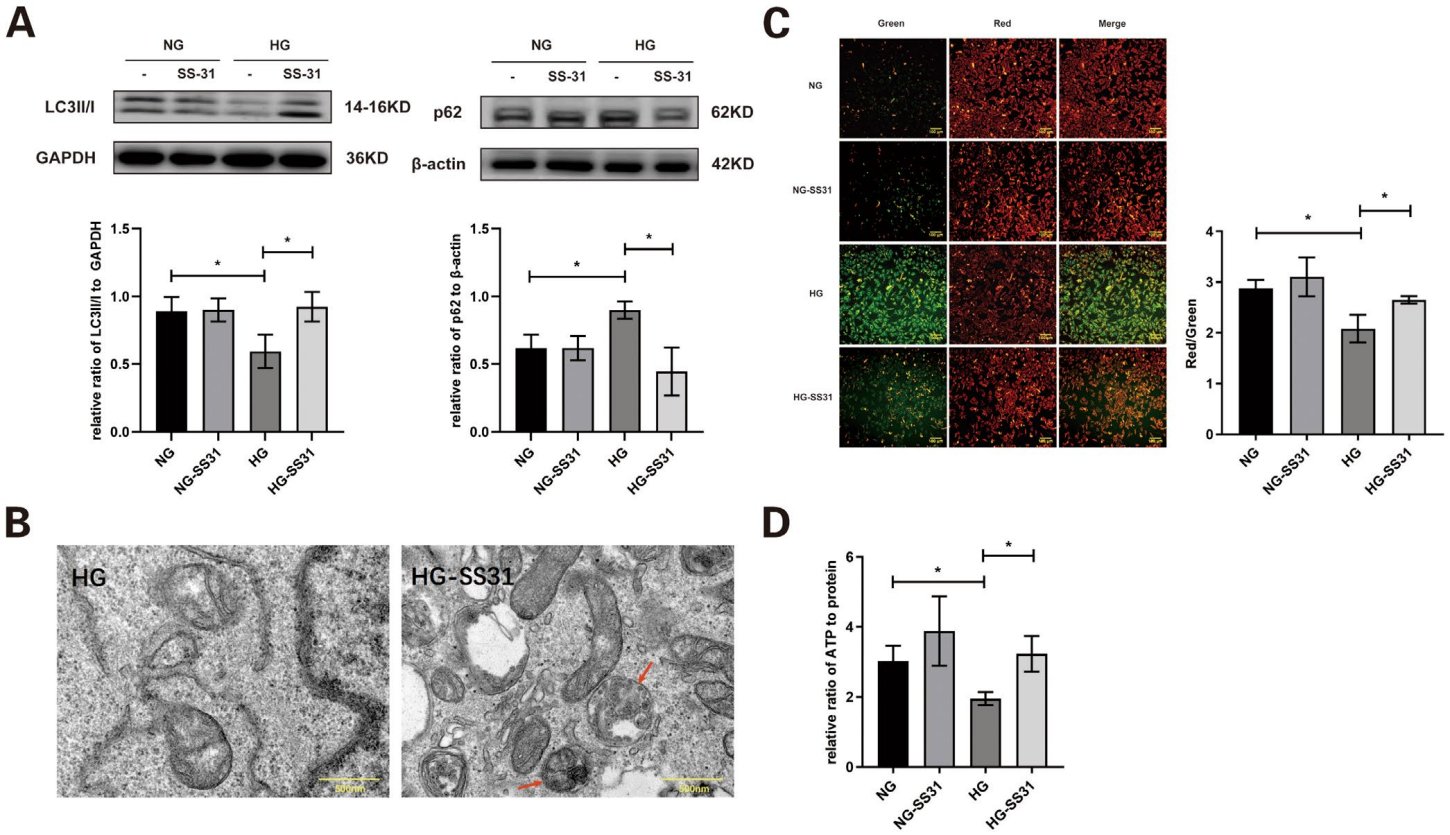
Effect of SS‐31 on mitochondria in HK‐2 cells. (A) LC3II/I and p62 levels of mitochondrial protein extracted from HK‐2 cells were assessed using western blotting. (B) Mitochondria and autophagosomes (indicated by red arrows) in HK‐2 cells were examined by transmission electron microscopy (magnification: 20,000×). (C) Mitochondrial membrane potential in HK‐2 cells was measured. (D) ATP concentration in HK‐2 cells was measured. SS‐31, Szeto‐Schiller 31; NG, normal glucose group; HG, high glucose group; *p < 0.05.

In in vitro studies, compared to the HG group in HK‐2 cells, LC3II/I level of mitochondrial proteins were significantly increased in cells treated with SS‐31, while p62 expression was significantly decreased (Figure 6A). Morphological changes such as swelling, fragmentation and disappearance of cristae were significantly improved, and mitochondrial autophagosomes were observed in SS‐31‐treated cells (Figure 6B). Additionally, both MMP and ATP concentrations were significantly increased following SS‐31 treatment (Figure 6C,D).

**FIGURE 7:**
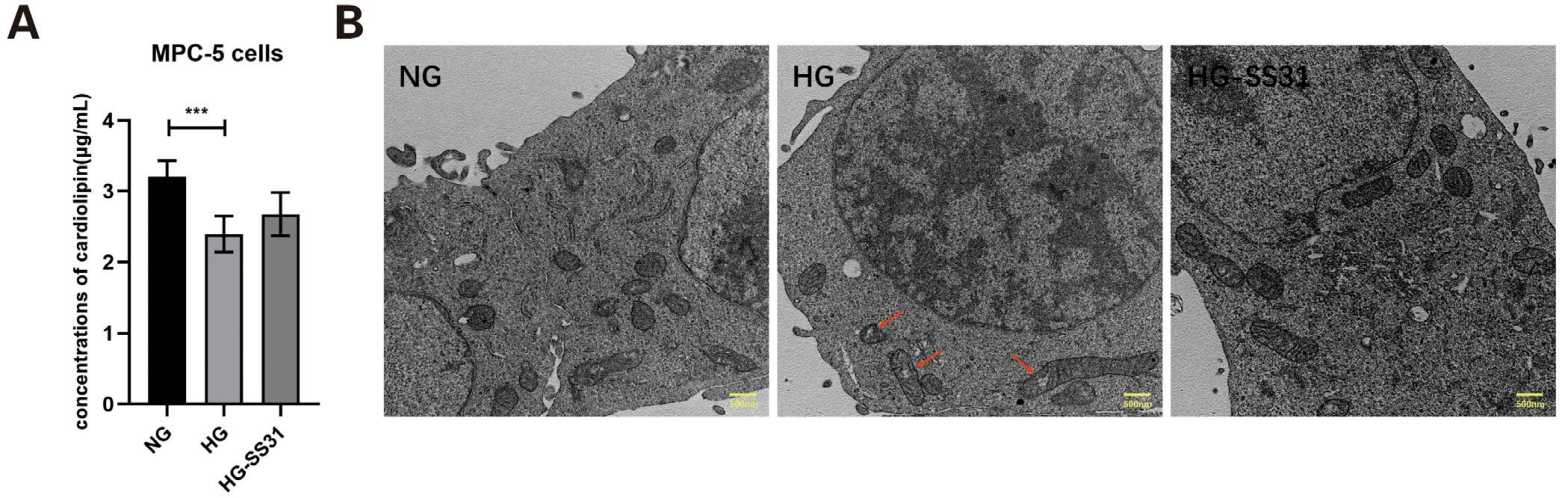
Effect of SS‐31 on CL and mitochondria in podocytes. (A) The concentration of cardiolipin in different groups was measured using ELISA. (B) Representative transmission electron microscopic image of normal mitochondria and damaged mitochondria (indicated by red arrow) in podocytes treated with NG, HG and HG+SS‐31 (magnification: 8000×). SS‐31, Szeto‐Schiller 31; NG, normal glucose group; HG, high glucose group; ***p < 0.001.

In order to clarify the expression of CL in glomerular cells, podocytes recognised as rich in mitochondria were selected as an example to observe the expression levels of CL and the changes in CL under HG stimulation and SS‐31 intervention. Results indicated that CL levels in podocytes were decreased under HG condition, and SS‐31 did not change the level of CL (Figure 7A). To further confirm the improvement of SS‐31 on mitochondrial damage, we used transmission electron microscope to observe mitochondria. We identified that increased vacuoles appeared in mitochondrial matrix in podocytes treated with HG, and SS‐31 could alleviate the mitochondrial damage profoundly (Figure 7B).

## 4 Discussion

The aim of this study was to understand the lipid‐related pathogenic mechanism of early‐stage DKD. The BKS *db/db* mouse model used in this study, which normally gets diabetes around 8 weeks and exhibits key characteristics similar to the human condition around 20 weeks—such as renal hypertrophy, glomerular enlargement, albuminuria and mesangial matrix expansion—is regarded as one of the most suitable models for DKD [19, 20, 21]. Starting from 12 weeks of age, the damage of renal proximal tubules was further confirmed in *db/db* mice in this study. FFA, LacCer and CL were identified as the most significantly altered lipids from diabetes to early diabetic nephropathy through dynamic observation of lipids changes in the renal cortex of *db/db* mice aged 8, 12, and 20 weeks.

In abnormal lipid metabolism, persistent hyperglycaemia and hyperinsulinemia play a crucial role in increasing FFA [22]. FFA are the preferred energy source for highly metabolic organs, including the kidney, where mitochondrial fatty acid β‐oxidation is the main source of ATP in healthy tubular epithelial cells [23]. Excessive accumulation of FFA disrupts cellular homeostasis and leads to lipotoxicity. A notable finding of this study was the abnormal accumulation of FFA in the renal cortex, not only in early‐stage DKD but also under diabetic conditions. This underscores the importance of reducing FFA levels to prevent kidney damage in diabetes.

Sphingolipids are closely associated with the development of DKD [24, 25]. This study examined ceramides and their derivatives, including GluCer, galactosylceramides (GalCer), LacCer and gangliosides. Previous research has reported an increase in LacCer levels in diabetic *db/db* mice [26]. The findings of this study not only align with previous reports but also reveal a significant increase in total LacCer in the renal cortex from diabetes to early‐stage DKD. This highlights LacCer's persistent role in lipotoxicity and DKD development. LacCer adversely affects the kidneys by increasing oxidative stress and generating advanced glycation end products [27, 28]. According to the sphingolipid metabolic pathway, ceramide can be converted to GalCer and GluCer through the addition of galactose and glucose, respectively, while LacCer, formed by adding galactose to GluCer, can be further converted into gangliosides [29]. Considering previous reports that ceramide, GluCer and gangliosides (especially monosialodihexosyl ganglioside, GM3) play roles in DKD development, the results of this study confirm a LacCer‐centric effect on DKD [30, 31, 32, 33, 34, 35]. Further research on lipid subclasses in different kidney cells and dynamic analyses of sphingolipid transformations would provide additional insights.

A most notable change of CL was observed in the renal cortex of *db/db* mice during early‐stage DKD in this study. Twenty‐two CL species increased significantly at 8 weeks but decreased notably at 12 weeks, and the total CL was significantly decreased at 12 weeks. A notable decrease in total CL was observed in the renal cortex during early‐stage DKD in this study. CL is a unique mitochondrial phospholipid composed of a skeleton formed by two phosphate molecules and three glycerol molecules, with four fatty acid molecules attached [36, 37]. Targeting the inner mitochondrial membrane, CL plays a crucial role in maintaining mitochondrial structure and function. Mitochondrial fragmentation has been observed even in the early stage of diabetic kidney injury, and mitochondrial dysfunction is linked to the development of DKD [38, 39, 40, 41]. Besides the well‐studied functions such as keeping mitochondrial electron transport chain, producing reactive oxygen species and participating in apoptosis, CL has also been found to participate in mitophagy as receptors by relocating to the mitochondrial outer membrane. CLS was reported to be one of the dependent ways of the externalisation of CL.

To investigate the role of CLS in CL‐mediated mitophagy in renal tubules under high glucose conditions, further research about CLS and the whole CL synthesis pathway was conducted in HK‐2 cells. In the CL synthesis pathway, the precursor glycerol 3‐phosphate undergoes acylation to form phosphatidic acid. Phosphatidic acid then condenses, catalysed by CDS, to produce cytidine diphosphate diacylglycerol. Cytidine diphosphate diacylglycerol is subsequently converted into phosphatidylglycerol phosphate by PGS. Finally, CLS catalyses the formation of nascent CL. The newly formed CL, lacking precise fatty acyl side chains, undergoes remodelling mediated by TAZ or monolysocardiolipin acyltransferase, resulting in mature CL with specific fatty acyl side chains [42, 43]. Among these proteins, both protein and mRNA expression of CLS were significantly reduced, and inhibition of CLS expression resulted in decreased CL levels in HK‐2 cells under HG stimulation. Conversely, overexpression of CLS promoted mitophagy, which was impaired under HG conditions, leading to a restoration of mitochondrial morphological integrity and improved mitochondrial function. The findings reveal that CLS influences CL‐related mitophagy in the renal proximal tubules, providing new insights into the pathogenesis of early DKD.

SS‐31, a mitochondrial‐targeting peptide, binds selectively to CL via electrostatic and hydrophobic interactions and forms a 1:1 molecular complex with CL in the mitochondrial inner membrane [44]. Although limited research has evaluated SS‐31 specifically for DKD, its protective effects have been documented. Hou et al. [45] demonstrated that SS‐31 offers protective benefits in DKD mice induced by uninephrectomy combined with streptozotocin injection through its antioxidant properties. Yang et al. [46] showed that SS‐31 reduces diabetic renal tubulointerstitial apoptosis by regulating mitochondrial dynamics in streptozotocin‐induced mice. Previous research with *db/db* mice has shown that SS‐31 protects against DKD by inhibiting mitochondrial fission and restoring renal superoxide production [47, 48]. This study extends those findings by highlighting SS‐31's role in promoting mitophagy in HK‐2 cells, thus providing a more comprehensive understanding of its renal protective mechanisms. In this study, SS‐31 didn't change the expression of CL both in vivo and in vitro studies. Unlike approaches that focus on increasing CL content, SS‐31 offers a distinct mechanism of action. SS‐31 has gained significant attention due to its high mitochondrial affinity and effective absorption, advantages that traditional mitochondrial‐targeting drugs lack [49, 50]. Analysing the mode of action between SS‐31 and CL in mitophagy in the future will provide theoretical basis for developing effective therapies for DKD.

The present study has some limitations. First, although differentially expressed lipids were identified during the progression of DKD, not all changes exhibited sustained statistical significance, though consistent trends were observed. To obtain more accurate information, future research could focus on precisely isolating and identifying these differentially expressed lipids and their subclasses for a more detailed analysis. Second, due to the specificity of lipid‐related mechanisms in cells and the lack of sensitive methods suitable for measuring renal intrinsic cells in vivo, we only analysed lipid changes of the whole renal cortex. Third, Ceramide is also an important lipid involved in LC3‐mediated mitophagy; however, this study only investigated the effects of CL on mitophagy. In the future, conducting research on ceramide‐related mitophagy and the possible interaction with CL would provide a more comprehensive elucidation of the mechanism of lipid‐related mitophagy. Based on current research findings, the prospects for future research include: (1) clarifying the mechanism of reduced expression of CLS in renal tubules under high glucose and searching for possible intervention targets for early DKD; (2) exploring the relevant mechanisms of decreased CL in podocyte injury and its possible crosstalk with renal tubular cells to comprehensively and deeply understand the function of CL in early DKD; (3) analysing the mode of action between SS‐31 and CL in mitophagy to provide theoretical basis for exploring targeted treatment to DKD.

## 5 Conclusions

The present results provide a comprehensive overview of lipid changes in the renal cortex, and CL is identified as one of the most significantly changed lipids from diabetes to early‐stage DKD. The reduction of CLS under high glucose conditions causes the impairment of CL‐related mitophagy by decreasing the synthesis of CL in proximal tubular cells. Additionally, the renal protective effect of mitochondrial CL‐targeted peptide SS‐31 was observed, and its mechanism of protecting the kidneys by activating mitophagy was demonstrated in renal proximal tubular cells. These findings offer valuable insights into the lipotoxicity‐related pathogenesis of early‐stage DKD and provide a promising therapeutic peptide for DKD treatment.

## Author Contributions

**Zhijie Li:** methodology (lead), writing – original draft (lead). **Hongmiao Wang:** data curation (equal), methodology (equal). **Nan Liu:** data curation (equal), methodology (equal). **Xiayuchen Lan:** data curation (equal), methodology (equal). **Ailing Xie:** methodology (equal), supervision (equal), validation (equal). **Ge Yuan:** methodology (equal), supervision (equal), validation (equal). **Bowen Li:** data curation (equal), methodology (equal). **Jiaxin Geng:** data curation (equal), methodology (equal). **Xiaodan Liu:** funding acquisition (lead), project administration (lead), writing – review and editing (lead).

## Conflicts of Interest

The authors declare no conflicts of interest.

## Supporting information


**Data S1.** Materials and Methods: Lipid extraction, Lipidomics analysis, Cell culture, Western blotting.

**Figure S1.** Representative transmission electron microscopic image of damaged mitochondria in proximal tubular cells in 12‐week *db/db* mice. Indicated by red arrow; magnification: 8000 ×.

**Figure S2.** Quality control of lipidomics. QC, quality control samples (aliquots of pooled sample).

**Figure S3.** General analysis of lipidomic profiles in kidney cortex of the diabetic *db/db* mice and wild‐type *db/m* mice. (A) Species detected in each 30 major lipids of kidney cortex; (B) the heatmap of total lipids in kidney cortex of the diabetic *db/db* mice and wild‐type *db/m* mice at the ages of 8, 12 and 20 weeks. *n* = 3 per group.

**Figure S4.** Differential expression of free fatty acids in kidney cortex between age‐matched diabetic *db/db* mice and wild‐type *db/m* mice at 8, 12 and 20 weeks. FFA, free fatty acids; *n* = 3 per group.

**Figure S5.** Differential expression of sphingolipids in kidney cortex between age‐matched diabetic *db/db* mice and wild‐type *db/m* mice at 8, 12 and 20 weeks. LacCer, lactosylceramides; GluCer, glucosylceramides; Cer, ceramides; *n* = 3 per group.

**Figure S6.** The heatmap of gangliosides in kidney cortex of 20‐week diabetic *db/db* and control *db/m* mice. *n* = 3 per group.

**Figure S7.** Differential expression of cardiolipin in kidney cortex between age‐matched diabetic *db/db* mice and wild‐type *db/m* mice at 8, 12, and 20 weeks. CL, cardiolipin; *n* = 3 per group.

**Figure S8.** The heatmap of cardiolipin in kidney cortex of 20‐week diabetic *db/db* and control *db/m* mice. CL, cardiolipin; *n* = 3 per group.

**Figure S9.** Effect of high glucose stimulation on key enzymes of the cardiolipin synthesis pathway in HK‐2 cells. (A) The protein level of CDS, PGS and TAZ in HK‐2 cells was quantified using western blotting. (B) The mRNA expression of CDS, PGS and TAZ in HK‐2 cells was assessed by RT‐PCR. CDS, cytidine diphosphate diacylglycerol synthase; PGS, phosphatidylglycerol synthase; TAZ, tafazzin. NG, normal glucose group; HG, high glucose group; *n* = 3, *p < 0.05.

**Figure S10.** Validation of the efficacy of CLS overexpression and knockdown treatment. (A) The protein level of CLS after CLS overexpression plasmid transfection was quantified using western blotting. (B) The protein level of CLS after CLS‐siRNA transfection was quantified using western blotting. (C) The mRNA expression of CLS after different CLS‐siRNA transfection was assessed by RT‐PCR. CLS, cardiolipin synthase; NC, normal glucose group; HG, high glucose group; CLS+, the CLS expression plasmid transfection group; siRNA, the CLS‐siRNA knockdown group; *n* = 3, *p < 0.05, **p < 0.01, ***p < 0.001.

## Data Availability

The data generated and analysed during the current study are available from the corresponding author upon reasonable request.
