# Supplementary material for: Renal Lipid Alterations From Diabetes to Early‐Stage Diabetic Kidney Disease and Mitophagy: Focus on Cardiolipin
Source: J Cell Mol Med. 2025 Feb 12;29(3):e70419. doi: 10.1111/jcmm.70419 (PMC11816159; doi:10.1111/jcmm.70419)
Supplement: Supplementary file 1 — Data S1. Materials and Methods: Lipid extraction, Lipidomics analysis, Cell culture, Western blotting. [file JCMM-29-e70419-s007.docx]

**DATA SUPPLEMENT**

**Renal lipid alterations from diabetes to early-stage diabetic kidney disease and mitophagy: Focus on cardiolipin**

Zhijie Li^1^, Hongmiao Wang^1^, Nan Liu^1^, Xiayuchen Lan^1^, Ailing Xie^1^, Ge Yuan^1^, Bowen Li ^2^, Jiaxin Geng ^2^, Xiaodan Liu^1^

^1^ Department of Nephrology, The First Hospital of China Medical University, Shenyang, Liaoning, China, 110001

^2^ LipidALL Technologies Company Limited, Changzhou, Jiangsu Province, China, 213022

Correspondence to:

Xiaodan Liu

Department of Nephrology

The First Hospital of China Medical University

155 North Nanjing Street

Shenyang, Liaoning, P.R. China, 110001.

Tel +86 13804023377

Fax +86 024 83282733

Email: [xdliu@cmu.edu.cn](mailto:xdliu@cmu.edu.cn)

Running title: Renal lipid alterations in diabetic kidney disease

**Materials and Methods**

**Lipid extraction**

Lipids were extracted from approximately 30 mg of frozen tissue using a modified version of the Bligh and Dyer method as previously described.^12^ Briefly, tissues were homogenized in 750 µL of chloroform: methanol: MilliQ H_2_O (3:6:1) (v/v/v). The homogenate was then incubated at 1500 rpm for 1h at 4°C. At the end of the incubation, 350 µL of deionized water and 250 µL of chloroform were added to induce phase separation. The samples were then centrifuged and the lower organic phase containing lipids was extracted into a clean tube. Lipid extraction was repeated once by adding 450 µL of chloroform to the remaining aqueous phase, and the lipid extracts were pooled into a single tube and dried in the SpeedVac under OH mode. Samples were stored at -80°C until further analysis.

**Lipidomics analysis**

Lipidomic analyses were conducted using an ExionLC-AD coupled with a Sciex QTRAP 6500 PLUS at LipidALL Technologies Company Limited.^13,14,15^ Separation of individual lipid classes of polar lipids by normal phase (NP)-HPLC was carried out using a TUP-HB silica column (i.d. 150x2.1 mm, 3 µm) with the following conditions: mobile phase A (chloroform: methanol: ammonium hydroxide, 89.5:10:0.5) and mobile phase B (chloroform: methanol: ammonium hydroxide: water, 55:39:0.5:5.5). MRM transitions were set up for comparative analysis of various polar lipids. Individual lipid species were quantified by referencing to spiked internal standards. d_9_-PC32:0(16:0/16:0), d_9_-PC36:1p(18:0p/18:1), d_7_-PE33:1(15:0/18:1), d_9_-PE36:1p(18:0p/18:1), d_31_-PS(d_31_-16:0/18:1), d_7_-PA33:1(15:0/18:1), d_7_-PG33:1(15:0/18:1), d_7_-PI33:1(15:0/18:1), C17-SL, d_5_-CL72: 8(18:2), Cer d18:1/15:0-d_7_, C12:0 Cer-1-P, d_9_-SM d18:1/18:1, C8-GluCer, C8-GalCer, d_3_-LacCer d18:1/16:0, Gb3 d18:1/17:0, d_7_-LPC18:1, d_7_-LPE18:1, C17-LPI, C17-LPA, C17-LPS, C17-LPG, d17:1 Sph, d17:1 S1P were obtained from Avanti Polar Lipids. GM3-d18:1/18:0-d_3_ was purchased from Matreya LLC. Free fatty acids were quantitated using d_31_-16:0 (Sigma-Aldrich) and d_8_-20:4 (Cayman Chemicals).

Glycerol lipids including diacylglycerols (DAG) and triacylglycerols (TAG) were quantified using a modified version of reverse phase HPLC/MRM.^14^ Separation of neutral lipids were achieved on a Phenomenex Kinetex-C18 column (i.d. 4.6x100 mm, 2.6 µm) using an isocratic mobile phase containing chloroform: methanol: 0.1 M ammonium acetate 100:100:4 (v/v/v) at a flow rate of 300 µL for 10 min. Levels of short-, medium-, and long-chain TAGs were calculated by referencing to spiked internal standards of TAG(14:0)_3_-d_5_, TAG(16:0)_3_-d_5_ and TAG(18:0)_3_-d_5_ obtained from CDN isotopes, respectively. DAGs were quantified using d_5_-DAG17:0/17:0 and d_5_-DAG18:1/18:1 as internal standards (Avanti Polar Lipids).

Free cholesterols and cholesteryl esters were analysed under atmospheric pressure chemical ionization mode on a Agilent1260-Sciex 5500 as described previously, using d_6_-cholesterol and d_6_-C18:0 cholesteryl ester (CE) (CDN isotopes) as internal standards.^15^

**Cell culture**

The human renal proximal tubular epithelial cell (HK-2 cell) line was purchased from the Shanghai Institute for Biological Sciences Cell Resource Center. The HK-2 cells were cultured in normal glucose Dulbecco’s Modified Eagle Medium/Nutrient Mixture F-12 (DMEM/F-12) that was supplemented with 10% fetal bovine serum (FBS; FSP500, ExCell). Normal glucose DMEM/F-12 was a 1:1 mixture of DMEM (11966025, Gibco, Grand Island, NY, USA) and Ham’s F-12 (11765054, Gibco, Grand Island, NY, USA) that contained 5.56 mmol/L glucose. The mouse podocyte Clone-5 cells (MPC-5 cells) were cultured in normal glucose (5.6 mmol·L^-1^ glucose) RPMI-1640 (Procell, China) which was supplemented with 10% fetal bovine serum (FBS; FSP500, ExCell), anti-Mycoplasma (1:200, Procell, China), 1% Penicillin-Streptomycin Solution (Procell, China). The cells were maintained at 37°C in a 5% CO_2_ incubator. Once 80% confluence was reached, the cells were harvested using the standard trypsin digestion procedure and passaged at a split ratio of 1:2.

**Western blotting**

Total protein was extracted with RIPA buffer (Beyotime, Shanghai, China) mixed with a protease inhibitor cocktail (Sigma-Aldrich, USA), then the extractions were cracked at 4°C for 30 min and centrifuged at 4°C for 20 min. The supernatants were mixed with loading buffer prior to being boiled at 100°C for 10 min. Equal amounts of protein samples were separated through SDS-PAGE and then transferred to PVDF membranes (Millipore, Burlington, MA, USA). Membranes were blocked with milk for 1 h before incubation with primary antibodies overnight at 4°C.
