# Supplementary figures and images for: Renal Lipid Alterations From Diabetes to Early‐Stage Diabetic Kidney Disease and Mitophagy: Focus on Cardiolipin
Source: J Cell Mol Med. 2025 Feb 12;29(3):e70419. doi: 10.1111/jcmm.70419 (PMC11816159; doi:10.1111/jcmm.70419)

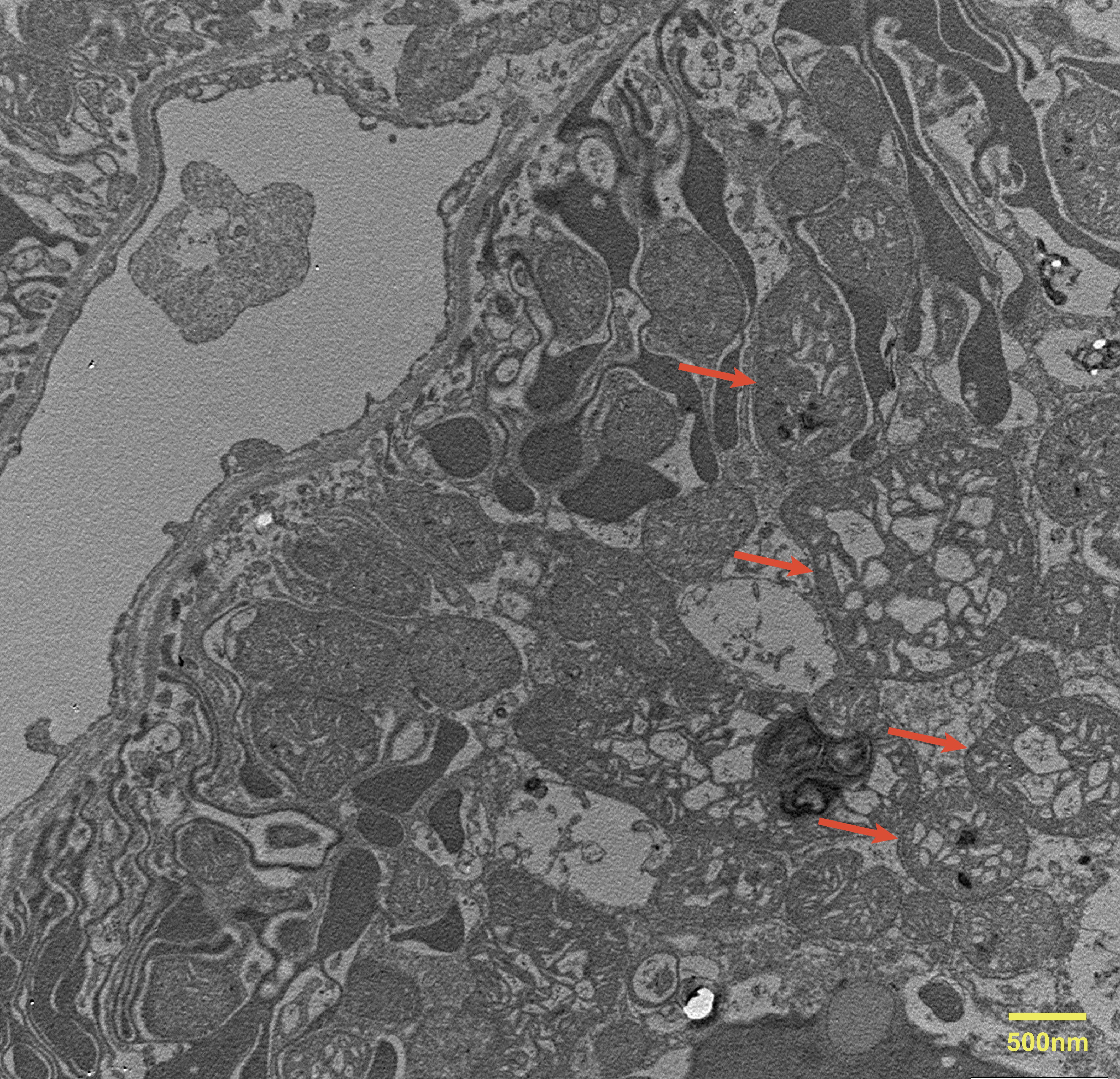

Supplement: Supplementary file 2 — Figure S1. Representative transmission electron microscopic image of damaged mitochondria in proximal tubular cells in 12‐week db/db mice. Indicated by red arrow; magnification: 8000 ×. [file JCMM-29-e70419-s010.jpg]

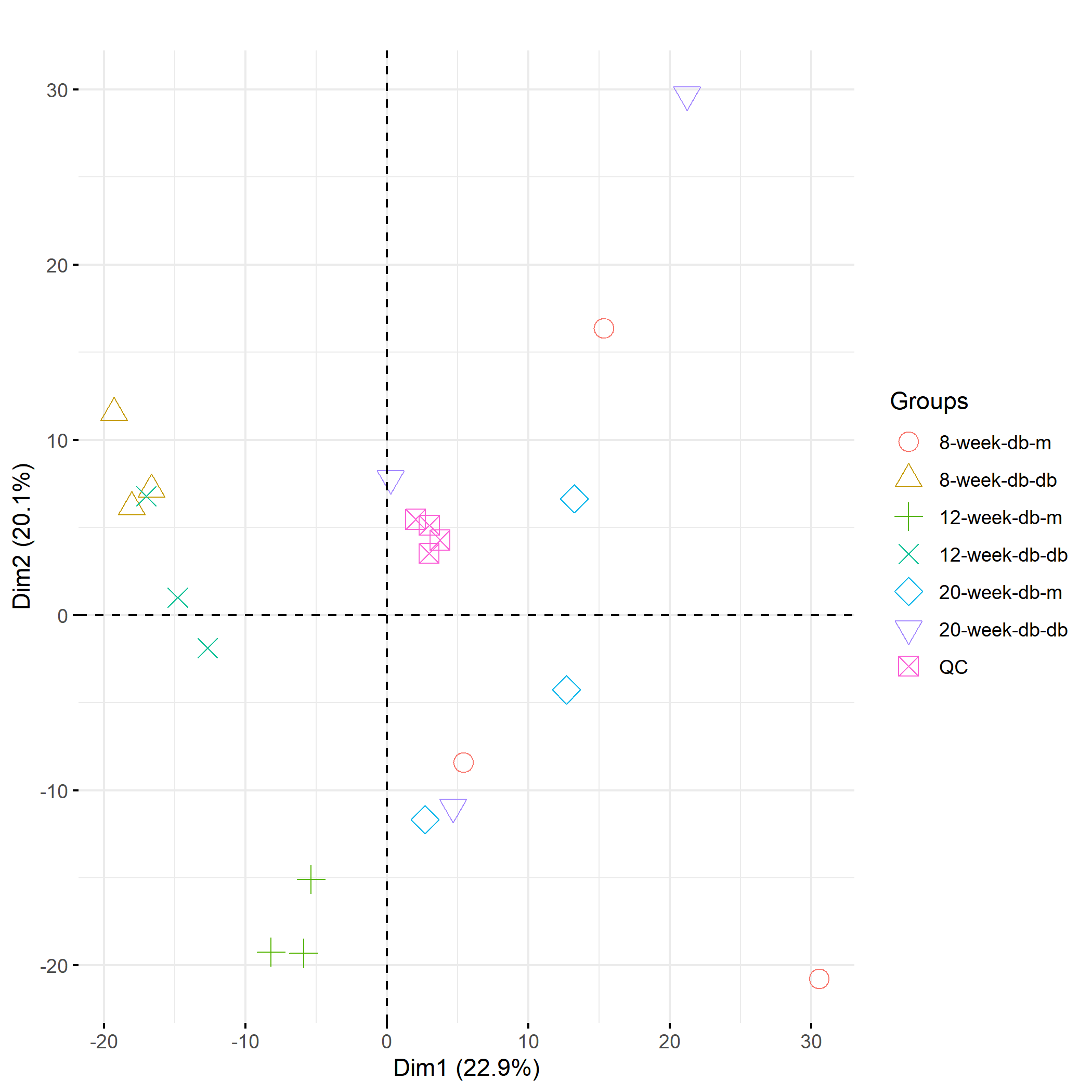

Supplement: Supplementary file 3 — Figure S2. Quality control of lipidomics. QC, quality control samples (aliquots of pooled sample). [file JCMM-29-e70419-s009.tiff]

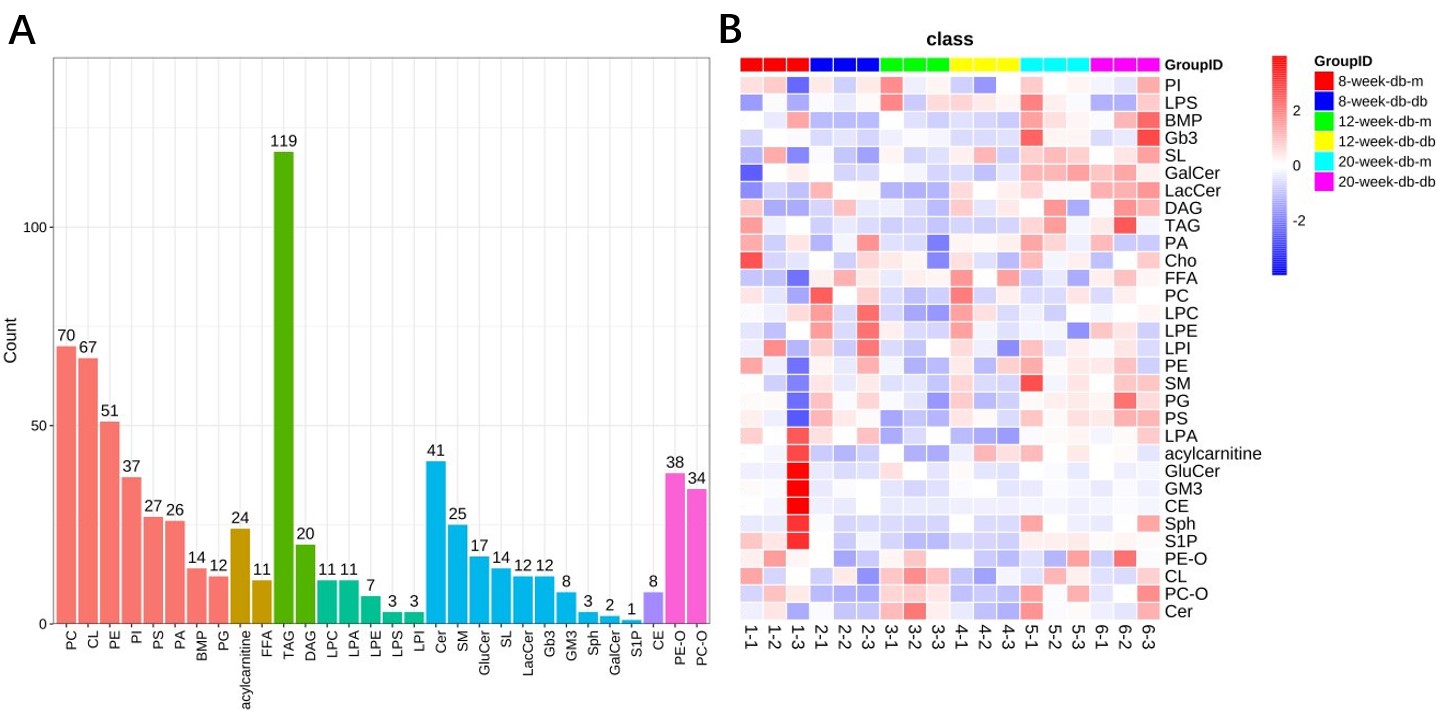

Supplement: Supplementary file 4 — Figure S3. General analysis of lipidomic profiles in kidney cortex of the diabetic db/db mice and wild‐type db/m mice. (A) Species detected in each 30 major lipids of kidney cortex; (B) the heatmap of total lipids in kidney cortex of the diabetic db/db mice and wild‐type db/m mice at the ages of 8, 12 and 20 weeks. n = 3 per group. [file JCMM-29-e70419-s004.jpg]

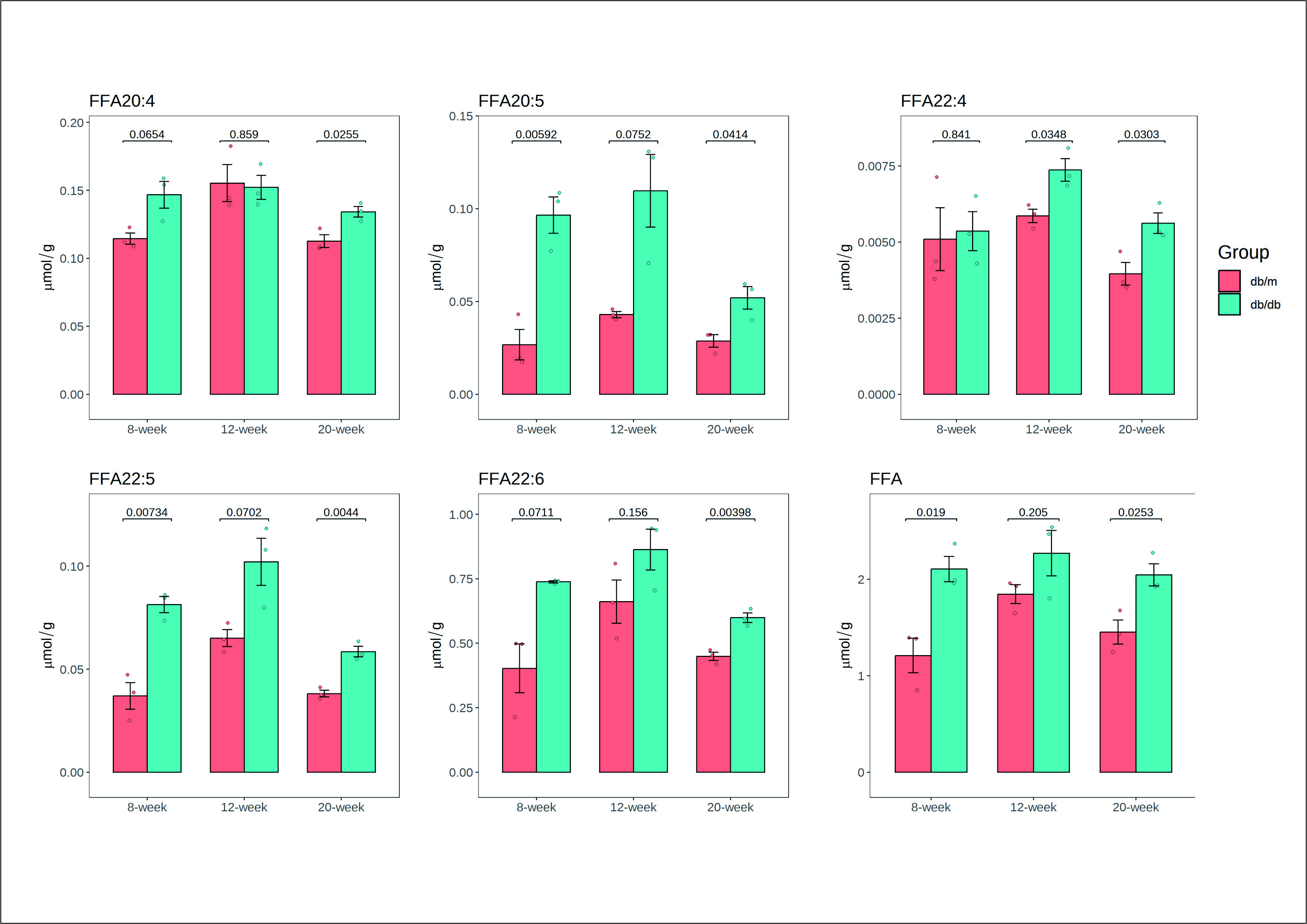

Supplement: Supplementary file 5 — Figure S4. Differential expression of free fatty acids in kidney cortex between age‐matched diabetic db/db mice and wild‐type db/m mice at 8, 12 and 20 weeks. FFA, free fatty acids; n = 3 per group. [file JCMM-29-e70419-s006.jpg]

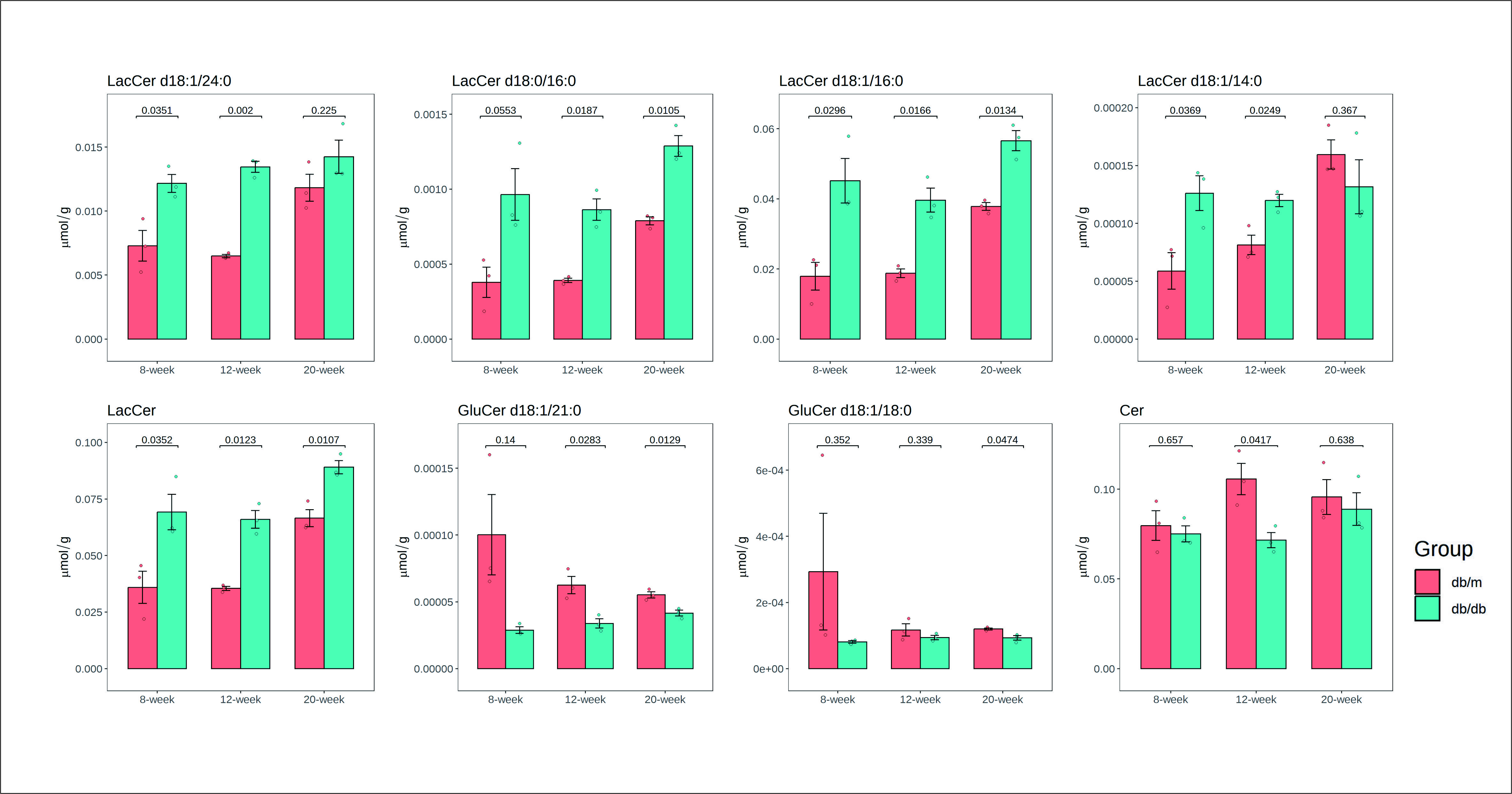

Supplement: Supplementary file 6 — Figure S5. Differential expression of sphingolipids in kidney cortex between age‐matched diabetic db/db mice and wild‐type db/m mice at 8, 12 and 20 weeks. LacCer, lactosylceramides; GluCer, glucosylceramides; Cer, ceramides; n = 3 per group. [file JCMM-29-e70419-s003.jpg]

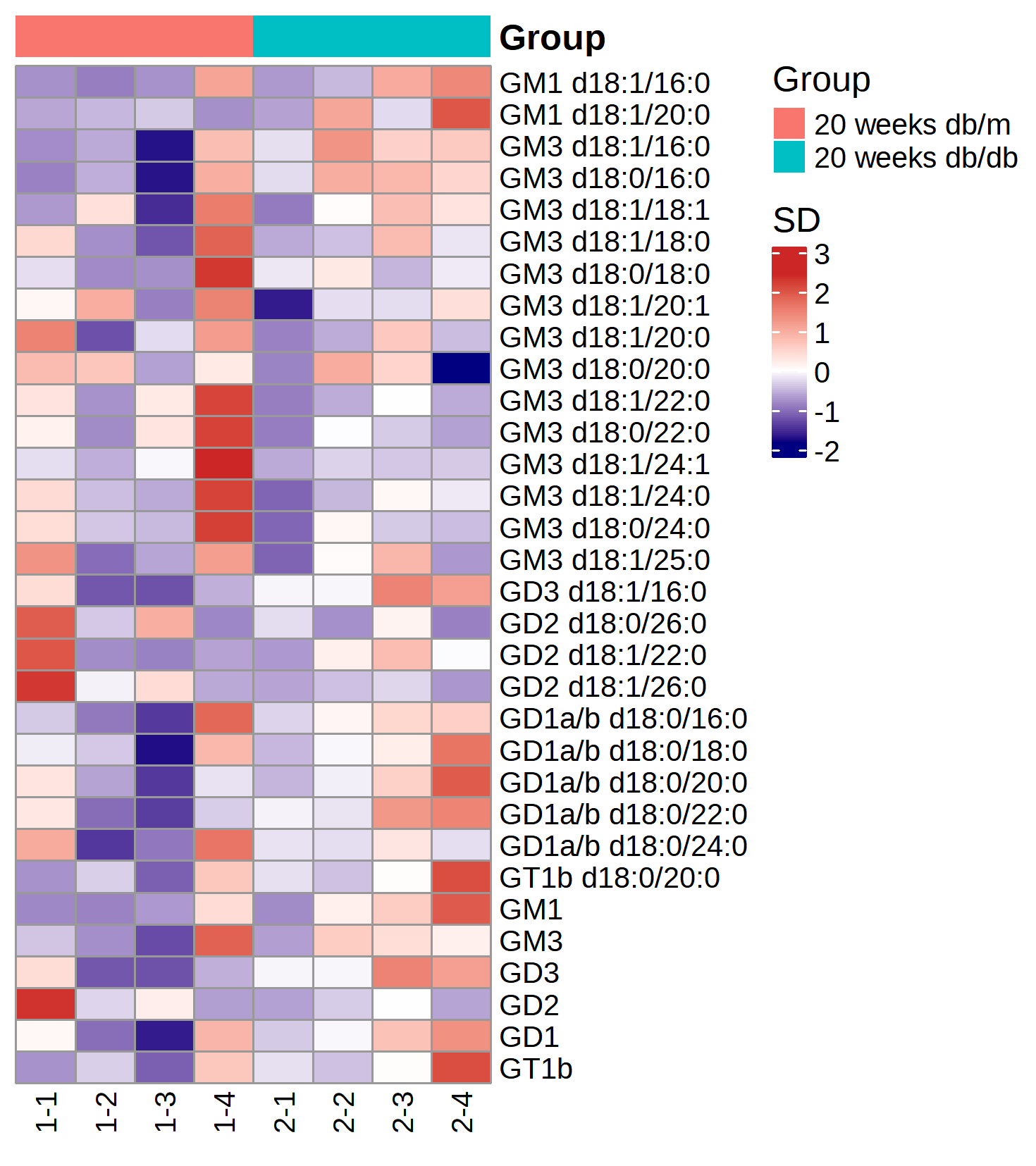

Supplement: Supplementary file 7 — Figure S6. The heatmap of gangliosides in kidney cortex of 20‐week diabetic db/db and control db/m mice. n = 3 per group. [file JCMM-29-e70419-s001.tiff]

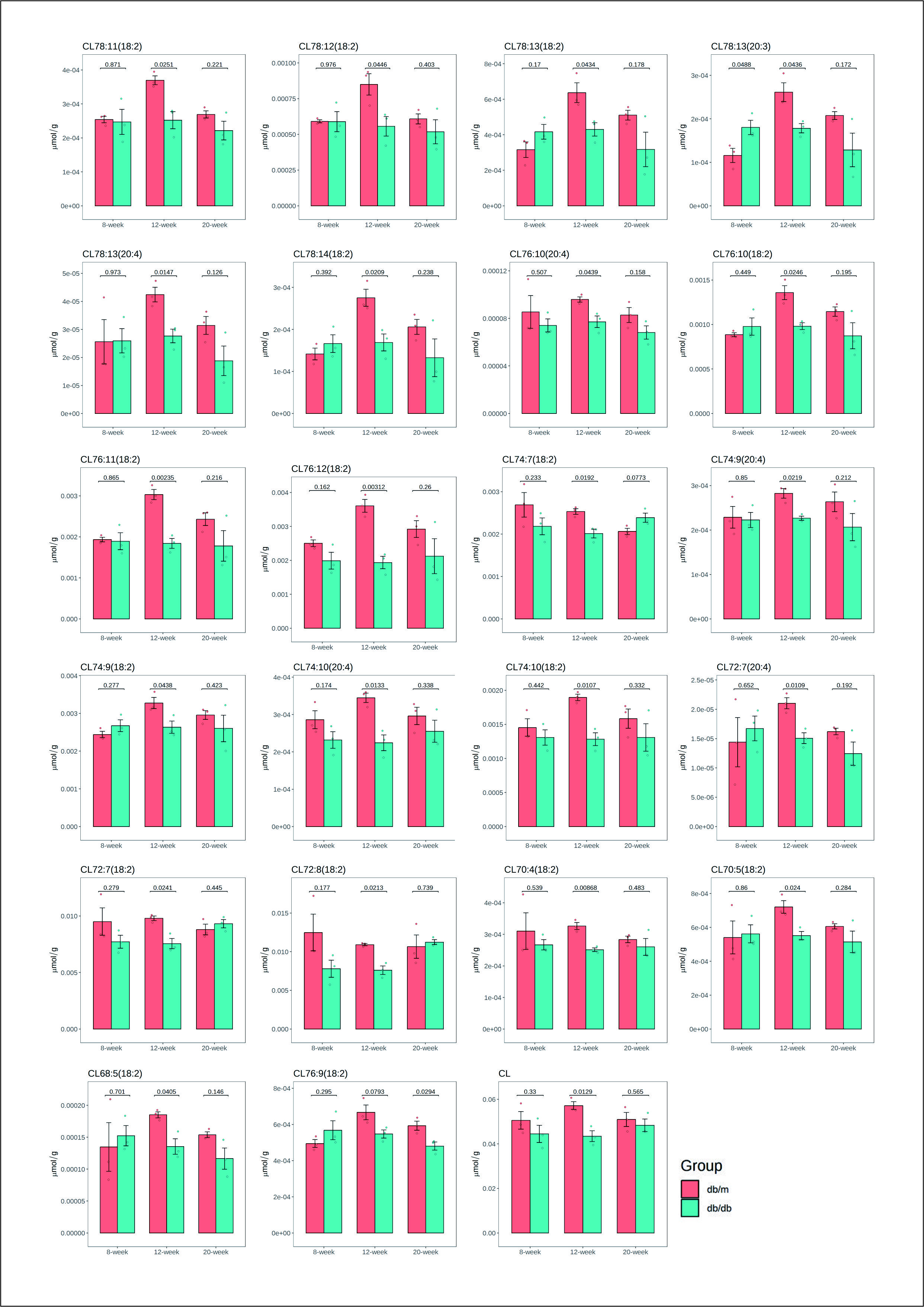

Supplement: Supplementary file 8 — Figure S7. Differential expression of cardiolipin in kidney cortex between age‐matched diabetic db/db mice and wild‐type db/m mice at 8, 12, and 20 weeks. CL, cardiolipin; n = 3 per group. [file JCMM-29-e70419-s008.jpg]

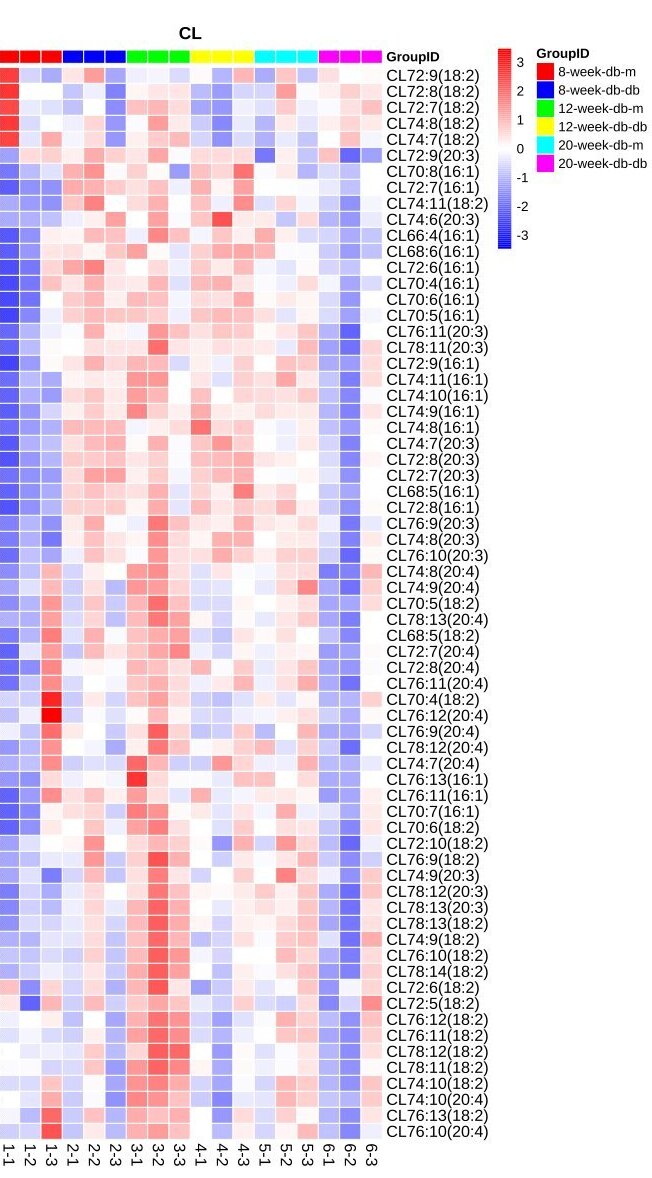

Supplement: Supplementary file 9 — Figure S8. The heatmap of cardiolipin in kidney cortex of 20‐week diabetic db/db and control db/m mice. CL, cardiolipin; n = 3 per group. [file JCMM-29-e70419-s002.jpeg]

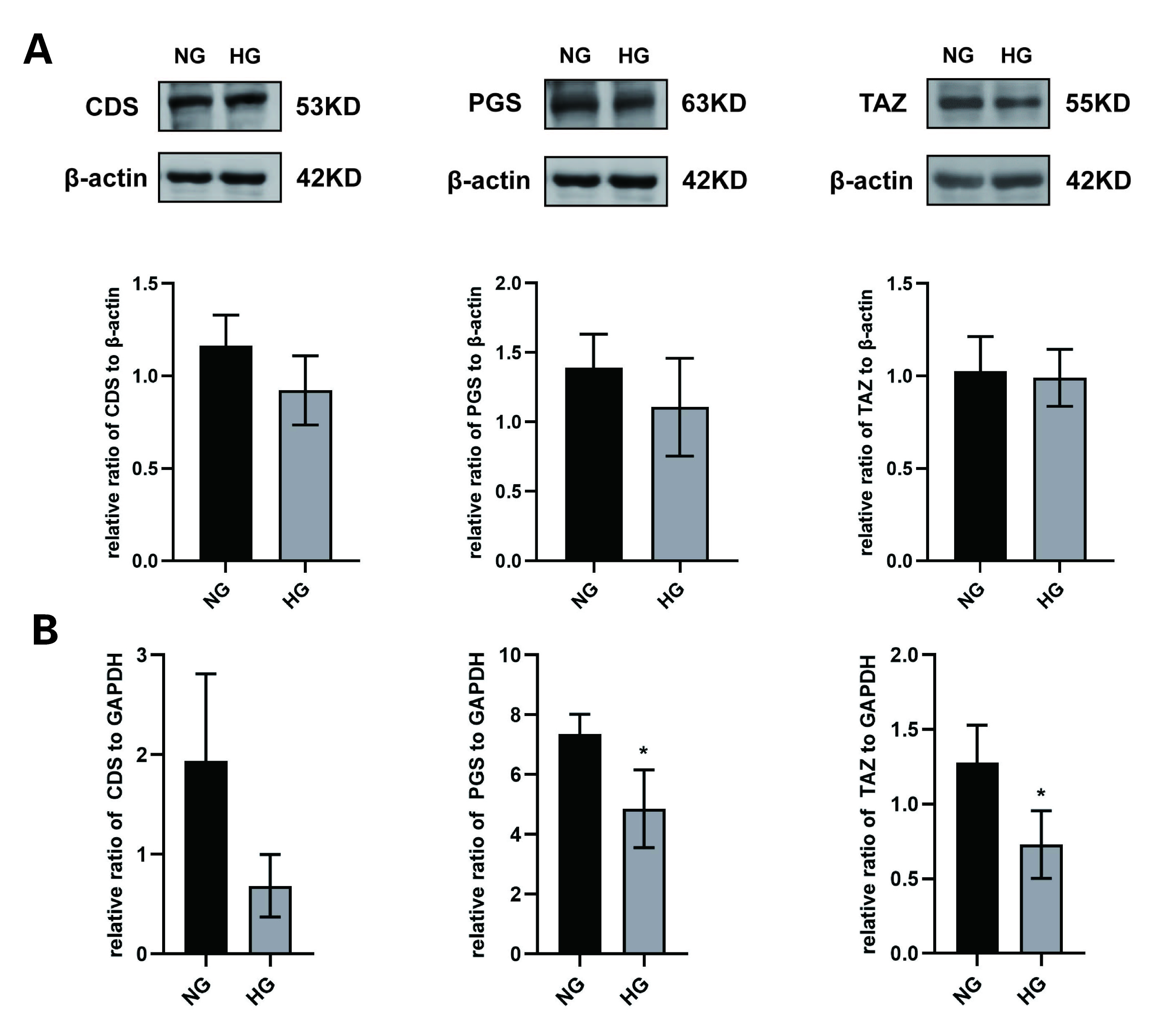

Supplement: Supplementary file 10 — Figure S9. Effect of high glucose stimulation on key enzymes of the cardiolipin synthesis pathway in HK‐2 cells. (A) The protein level of CDS, PGS and TAZ in HK‐2 cells was quantified using western blotting. (B) The mRNA expression of CDS, PGS and TAZ in HK‐2 cells was assessed by RT‐PCR. CDS, cytidine diphosphate diacylglycerol synthase; PGS, phosphatidylglycerol synthase; TAZ, tafazzin. NG, normal glucose group; HG, high glucose group; n = 3, *p < 0.05. [file JCMM-29-e70419-s005.jpg]

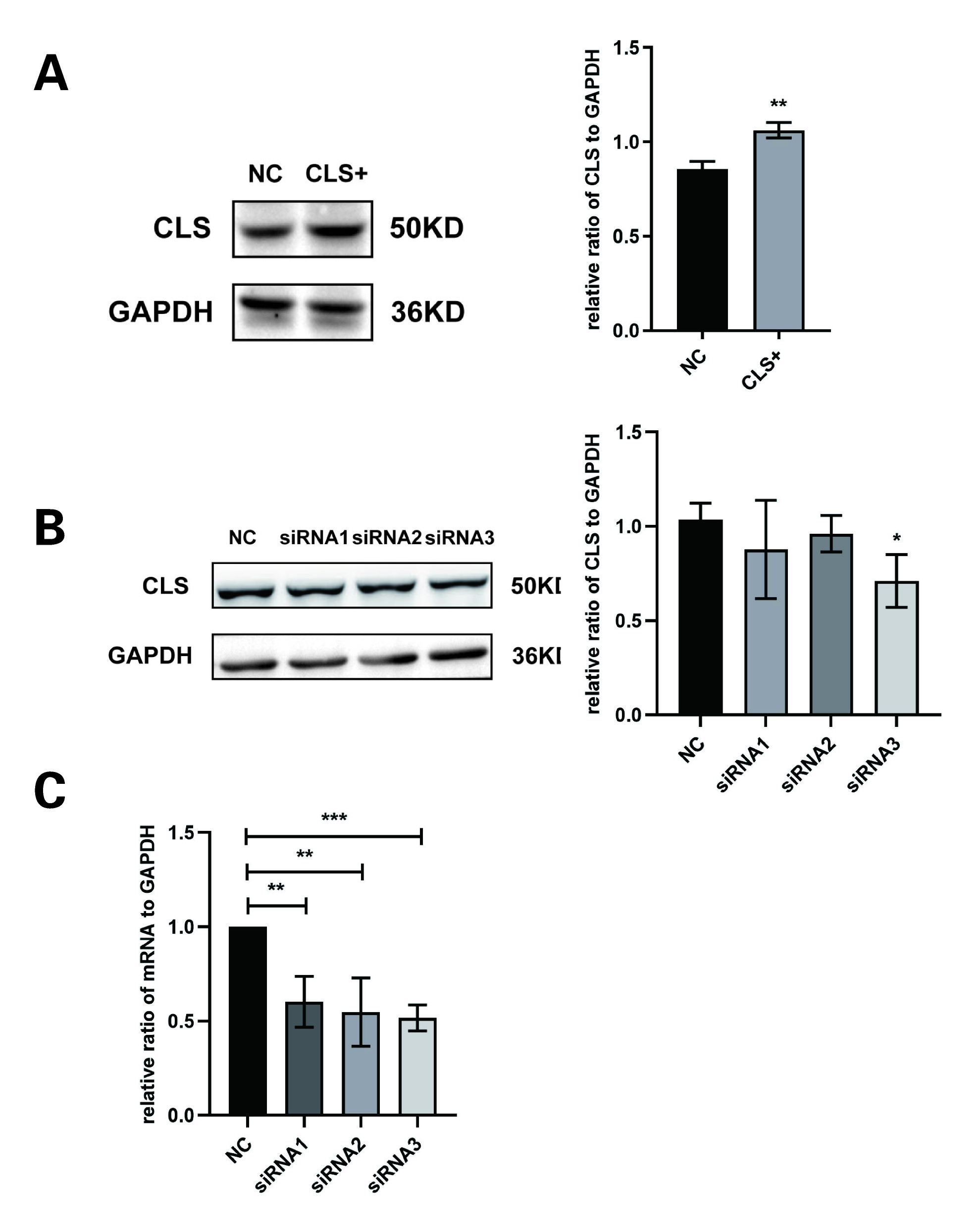

Supplement: Supplementary file 11 — Figure S10. Validation of the efficacy of CLS overexpression and knockdown treatment. (A) The protein level of CLS after CLS overexpression plasmid transfection was quantified using western blotting. (B) The protein level of CLS after CLS‐siRNA transfection was quantified using western blotting. (C) The mRNA expression of CLS after different CLS‐siRNA transfection was assessed by RT‐PCR. CLS, cardiolipin synthase; NC, normal glucose group; HG, high glucose group; CLS+, the CLS expression plasmid transfection group; siRNA, the CLS‐siRNA knockdown group; n = 3, *p < 0.05, **p < 0.01, ***p < 0.001. [file JCMM-29-e70419-s011.jpg]
